# Isoniazid–Saccharin Salts: Synthesis, Structural Aspects, Thermal Properties and Spectroscopic Characterization

**DOI:** 10.3390/molecules31122187

**Published:** 2026-06-22

**Authors:** Rezvan Mohammadi, Ayberk Yilmaz, Nihal Sarier, José António Paixão, Gulce Ogruc Ildiz, Rui Fausto

**Affiliations:** 1 Spectroscopy@IKU, IKU-SPECTRA Molecular Sciences and Spectroscopy Applied Research Center, Istanbul Kultur University, Atakoy Campus, Bakirkoy, Istanbul 34156, Türkiye; sp.rezvanm@iku.edu.tr (R.M.); ayberk@istanbul.edu.tr (A.Y.); n.sarier@iku.edu.tr (N.S.); g.ogruc@iku.edu.tr (G.O.I.); 2 Department of Physics, Faculty of Sciences and Letters, Istanbul Kultur University, Atakoy Campus, Bakirkoy, Istanbul 34156, Türkiye; 3 Department of Physics, Science Faculty, Istanbul University, Vezneciler, Istanbul 34118, Türkiye; 4 Department of Civil Engineering, Faculty of Engineering, Istanbul Kultur University, Atakoy Campus, Bakirkoy, Istanbul 34156, Türkiye; 5 CFisUC, Department of Physics, University of Coimbra, 3004-516 Coimbra, Portugal; jap@fis.uc.pt; 6 CQC–IMS, Department of Chemistry, University of Coimbra, 3004-535 Coimbra, Portugal

**Keywords:** isoniazid–saccharin salts, 2-[2-(pyridine-4-carbonyl)hydrazine-1-carbonyl]benzene-1-sulfonamide, single-crystal X-ray diffraction, infrared and Raman spectroscopies, DSC, DFT, fully periodic calculations, Hirshfeld analysis

## Abstract

This investigation focused on isoniazid (INH)—saccharin (SAC) salts. One hydrate and one anhydrous INH-SAC salt form were synthesized and characterized spectroscopically by Raman and infrared spectroscopy. Solvent (methanol, acetone, acetonitrile)-assisted synthesis in the presence of water, or in water, resulted in production of the monohydrated form of the salt (MH: (INH+H)^+^/(SAC–H)^−^.H_2_O). The anhydrous form (A: (INH+H)^+^/(SAC–H)^−^) was obtained using the same synthesis method but in the absence of water or, together with the hydrate, in the presence of traces of water. Differential scanning calorimetry studies revealed that the hydrate can be converted into the anhydrous form of the salt upon heating, with the latter melting at a *T*_m_ (onset) of 131.7 ± 0.5 °C. Melting was followed by a reaction between isoniazid and saccharin leading to saccharin ring opening and formation of a new covalent hydrazide–amide derivative, *via* nucleophilic acyl substitution at the saccharin carbonyl. The newly formed adduct, 2-[2-(pyridine-4-carbonyl)hydrazine-1-carbonyl] benzene-1-sulfonamide, melts at *T*_m_ (onset) = 204.4 ± 0.5 °C. The crystal structures of the hydrate and of the anhydrous form were determined by single-crystal X-ray diffraction, and the dominant intermolecular interactions in the crystalline INH-SAC salts were evaluated using Hirshfeld surface analysis. To complement the experimental results, density functional theory (DFT) calculations were performed both on relevant isolated structural units and on the two salts, employing fully periodic DFT methods.

## 1. Introduction

Isoniazid (INH) is an antibiotic widely used for the treatment of tuberculosis (TB), a disease that remains a major global health challenge [1,2,3,4,5,6,7]. In addition to its therapeutic use, isoniazid is also employed for TB prophylaxis in high-risk populations, including individuals with HIV [8,9,10]. Its mechanism of action involves inhibition of mycolic acid synthesis (an essential component of the *Mycobacterium tuberculosis* bacterial cell wall), ultimately leading to bacterial cell death [11,12,13]. As a first-line anti-TB drug, it is typically administered together with other antitubercular agents in fixed-dose combination (FDC) to reduce the risk of resistance development [14,15,16]. However, it has been shown that in the FDC tablet formulations, INH can undergo easier degradation due to drug–drug interactions [17,18]. For example, the presence of other anti-TB drugs, such as pyrazinamide and ethambutol, was found to accelerate the degradation of INH [19,20]. In addition, although INH does not show solubility/dissolution rate issues (it is a BSC class I drug) [18,21], it presents challenges related to hepatotoxicity, metabolic formation of reactive species, and potential mutagenicity, in addition to susceptibility to oxidation [22]. Consequently, improving the solid-state properties of INH and searching for safe solutions for INH’s limited stability in the FDC formulations have recently attracted much interest [19,23,24].

In the present investigation we report on novel isoniazid–saccharin (INH-SAC) salts. Saccharin is most known as one of the earliest artificial sweeteners, extensively utilized worldwide as a sugar substitute [25,26,27]. It is a highly water-soluble, well-established GRAS (“Generally Recognized As Safe”) excipient that has been shown to be able to improve bioavailability and physicochemical properties of several APIs [28,29]. Using a saccharin salt with isoniazid in a pharmaceutical formulation for isoniazid delivery offers important advantages mainly related to palatability and patient compliance as well as stability. Isoniazid has a distinctly bitter taste, which can reduce adherence, especially in pediatric or long-term tuberculosis treatments. Saccharin, being a high-intensity sweetener, helps mask this bitterness, making oral formulations more acceptable to patients. In addition, salt formation with saccharin can improve the organoleptic properties of the drug without compromising its therapeutic activity. It may also provide some benefits in terms of solid-state properties, such as improved crystallinity and handling during manufacturing. Overall, the isoniazid–saccharin dual system has the potential to enhance patient acceptability and support better compliance in treatment regimens. Our selection of the saccharin coformer was also motivated by the fact that, despite historical safety controversies, saccharin has been deemed safe by various health authorities, including the FDA [30,31,32], its extensive consumption has started to raise environmental concerns [33] due to its persistence and limited biodegradability. Consequently, its upcycling through association with INH or other bioactive substances [34] offers an appealing strategy to convert saccharin waste into high-value pharmaceutical materials, thus supporting circular-economy objectives while potentially improving drug performance.

A monohydrate and an anhydrous INH-SAC salt form (MH, A) have been synthesized in the present study, characterized spectroscopically by Raman and infrared spectroscopy, and investigated by differential scanning calorimetry (DSC) and thermogravimetric analysis (TGA). The crystal structures of the hydrate and the anhydrous form were determined by single-crystal X-ray diffraction (SC-XRD), and the dominant intermolecular interactions in the crystalline materials were further evaluated using Hirshfeld surface analysis [35,36,37,38,39,40]. To complement the experimental results, DFT calculations were performed on relevant INH/SAC isolated structural units, as well as on the MH and A crystals. In the latter case, fully periodic DFT calculations were employed.

## 2. Results and Discussion

### 2.1. Synthesis

**Figure 1:**
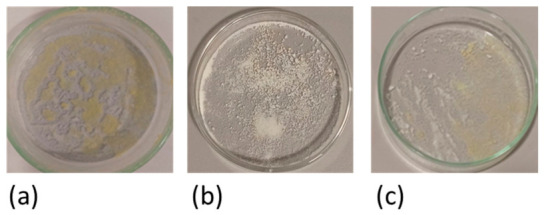
Crystalline materials obtained in the synthesis performed using different solvents: (**a**) water, (**b**) acetonitrile dried through molecular sieves (this experiment was performed under an inert atmosphere), and (**c**) acetonitrile containing traces of water (similar results were obtained when methanol and acetone containing traces of water were used as solvent).

Different variants of the synthetic procedure were used to prepare the INH-SAC salts. INH and SAC were initially ground together in an equimolar ratio (0.154 g INH and 0.206 g SAC) to guarantee that the initial reactants were uniformly dispersed prior to the commencement of the chemical interaction facilitated by the solvent. One milliliter of the chosen solvent was then added to the solid amalgamation, followed by a second addition of 1.0 mL of solvent, after 5 min incubation. The mixture was subjected to vigorous agitation and subsequently permitted to rest for an additional 5 min, after which a third 1.0 mL aliquot of solvent was added. The resultant solution was then left at room temperature until solvent evaporation. The solvents used were (a) water, (b) acetonitrile dried through molecular sieves (this experiment was performed under an inert atmosphere and in the presence of a Bruker desiccant canister), and (c) methanol, acetone and acetonitrile containing traces of water. A single yellowish crystalline phase was recovered from water, while a white crystalline material was observed as the result of the experiment performed using molecular sieve-dried acetone under an inert atmosphere. The remaining synthetic procedures yielded a mixture of both yellow and white crystalline phases (Figure 1). Examination under a microscope revealed that both yellow and white crystals exhibited a platelet-like habit. As described in detail in the next sections, subsequent analyses of the two obtained crystalline phases revealed that the yellow material corresponds to the monohydrate INH-SAC salt (MH: (INH+H)^+^/(SAC–H)^−^.H_2_O), and the white phase to the anhydrous INH-SAC salt form (A: (INH+H)^+^/(SAC–H)^−^).

### 2.2. XRD Results

**Figure 2:**
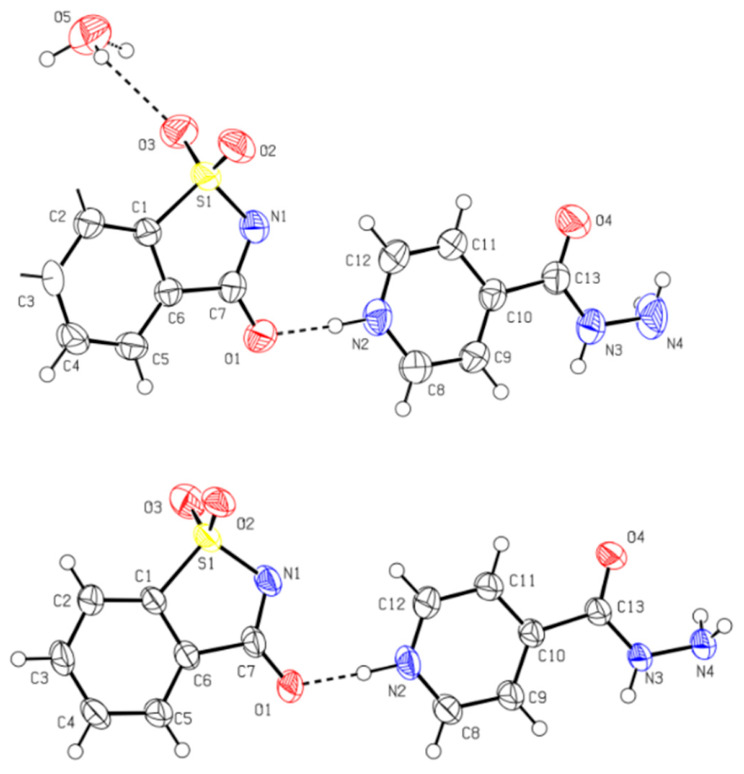
ORTEP drawing of the molecular structure of MH (**top**) and A (**bottom**) showing the atom numbering scheme. Displacement ellipsoids are drawn at the 50% probability level. The N–H···O hydrogen bonds are depicted as dashed lines. The water molecule in MH has one of its H atoms disordered over two alternate positions.

The two different synthesized crystalline phases were subjected to both SC-XRD and PXRD analysis. The yellow crystalline phase was shown to correspond to the INH–SAC monohydrate salt (MH). The MH structure is triclinic, belonging to the centrosymmetric space group *P*-1 (No. 2) with *a* = 7.3442(4) Å, *b* = 8.0527(4) Å, *c* = 12.5571(6) Å, *α* = 80.968(2)°, *β* = 83.032(2)°, *γ* = 84.994(2)°, *V* = 726.25(6) Å^3^ and two formula units ((INH+H)^+^/(SAC–H)^−^.H_2_O) per unit cell (*Z* = 2 and *Z*′ = 1) at 300(1) K (Table 1). The contents of the asymmetric unit of MH are shown in Figure 2. The observed location of the H atoms firmly establishes the presence of saccharin as an anion and isoniazid as a cation, linked together by a strong N2–H···O1 bond [N2^…^O1: 2.642(2) Å; ∠N2–H^…^O1: 177(2)°]. The structure contains one water molecule per formula unit, which has one of its H atoms involved in a hydrogen bond established with one of the O atoms of the saccharin SO_2_ group, with the other H atom being disordered over two alternate positions with close equi-occupancy. The crystal structure of MH contains no further solvent-accessible voids.

In the salt, the INH cation, (INH+H)^+^, adopts a conformation similar (only with a slightly more planar heavy-atom skeleton) to that of the INH molecule’s most stable isolated form [41,42] and in the neat INH’s most stable polymorph (polymorph 1) [43,44]. In the isolated INH molecule, the O=C–N–N, C–C–C=O, and C–N–N–H dihedral angles are −6.0°, 153.8°, and 82.2°/−32.6°, respectively (DFT/B3LYP/6-311++G(d,p) calculated values [42]) and in neat INH polymorph 1, they are −5.5(2)°, 160.5(2)°, and 85(1)°/−26(1)° [43,44]. In the (INH+H)^+^/(SAC–H)^−^.H_2_O hydrated salt, the corresponding O=C–N–N, C–C–C=O and C–N–N–H dihedral angles are 0.4(3)°, 9.1(3)°, and −79º/23(2)°. This molecular conformation is stabilized by an intramolecular NH_2_···O contact, with a N···O distance of 2.733(3) Å, while the second hydrogen atom of the NH_2_ group participates in a weaker hydrogen bond with the water molecule (N···O_w_ distance of 3.136(3) Å) (Table S7).

The white crystalline phase was identified as the anhydrous form of the INH-SAC salt (A: (INH+H)^+^/(SAC–H)^−^). The material crystallizes in the monoclinic system, space group *P*2_1_/*c*, with *a* = 11.3749(3) Å, *b* = 7.9243(2) Å, *c* = 15.3365(4) Å, *α* = *β* = 98.636(2)°, *V* = 1366.73(6) Å^3^ and *Z* = 4, *Z*′ = 1. In this crystal, (INH+H)^+^ assumes a similar conformation as in MH and as in INH in neat INH crystalline polymorph 1 (see Figure 2), with the O=C–N–N, C–C–C=O and C–N–N–H dihedral angles being −2.4(4)°, 171.3(3)°, and 66(2)°/−46(2)°, and the intramolecular NH_2_···O distance being 2.764(3) Å, i.e., slightly longer than that in MH.

In the crystalline structure of both compounds, the INH and SAC rings are almost coplanar (angle between the normals to the least-squares planes is 12.13(6)° in MH and 9.57(7)° in A).

**Figure 3:**
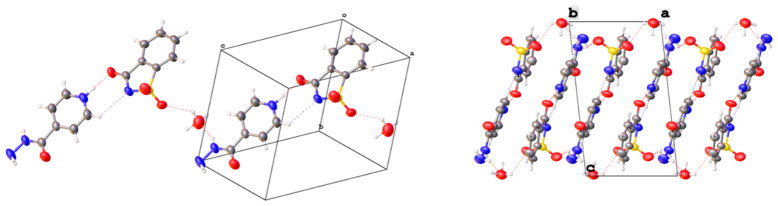
(**left**) Main hydrogen-bond motif present in the crystalline structure of MH, with the water molecule bridging (INH+H)^+^/(SAC–H)^−^ units *via* O–H··O and N–H···O bonds. Short contacts involving the disordered H atoms are not shown in the picture. (**right**) View of the crystal structure along the crystallographic *b*-axis.

In the MH crystal, the hydrazide N–H group of the cation acts as a proton donor towards the NH_2_ group of a neighbor cation forming a ring of $R_{2}^{2}\left(6\right)\;$topology, while both hydrogen atoms of the cation NH_2_ group participate in N–H^…^O bonds with SO_2_ groups of neighboring saccharin anions (N···O distances: 3.042(3) and 3.169(3) Å). In turn, the water molecule acts as a bridge element between (INH+H)^+^/(SAC–H)^−^ units, forming zig-zag chains (Figure 3). These chains are further interconnected by the hydrogen bonds involving the NH_2_ group of the cation and the SO_2_ group of the anion, resulting in a 3D network.

**Figure 4:**
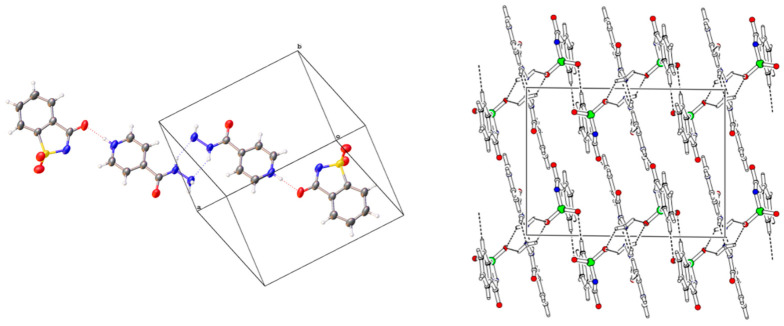
(**left**) Main hydrogen-bond motif present in the crystalline structure of A, with (INH+H)^+^/(SAC–H)^−^ units bound by short N–H···O hydrogen bonds that are further connected in chains by longer N–H···N interactions. (**right**) View of the structure of A along the crystallographic *b*-axis. The chains are arranged in layers that are interconnected by N–H···O hydrogen bonds, where the protons of the NH_2_ group are donated towards one of the O atoms of the SO_2_ groups of neighbor chains.

In the anhydrous A form, molecular packing is also mainly dictated by an extensive hydrogen bonding network. The short N2–H2···O1 bond (2.605 Å) connects the cations and anions in a pattern similar to that found in MH, and these units are further connected in chains via a longer (2.886 Å) N3–H3···O4 bond as depicted in Figure 4 (left panel). These chains are stacked in layers in the crystalline structure, the amino NH_2_ group acting as a proton donor towards the O atoms of the SO_2_ groups of saccharin anions of neighbor chains as depicted in the right panel of Figure 4.

**Figure 5:**
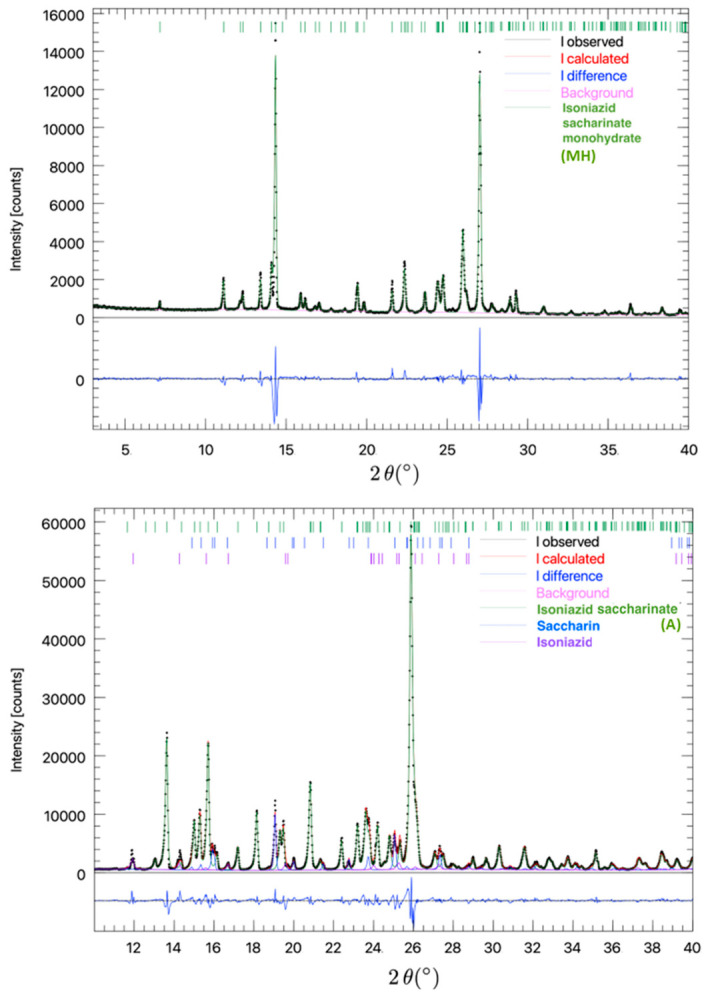
Measured and calculated (from Rietveld analysis) powder diffraction patterns for MH (**top**) and A (**bottom**). The blue line at the bottom of each panel represents the difference between the observed and calculated intensities.

In order to check the purity in bulk of the two compounds, X-ray diffraction patterns were collected in powder samples using Cu Kα radiation (Figure 5).

The PXRD data of MH shows a very good match with the calculated pattern of the structure determined by single-crystal XRD, without any extra Bragg peaks of extraneous phases (Figure 5, upper panel). Rietveld refinement, where all structural parameters were kept fixed to those determined from SCXRD and only those related to peak profile (particle size/strain) broadening, texture and background were refined, converged to *R*_p_ = 10.97% and *R*_wp_ = 10.72%.

The PXRD analysis of the used anhydrous sample also gave a good match with the pattern predicted from the structure determined from the single-crystal XRD data, with only a few minor extra peaks that could be assigned to free saccharin and isoniazid. Rietveld refinement of the data using the known structures was used to quantify the three substances in the powder. Similarly to the previous case, in the refinement all structural parameters (atomic positions and displacement factors) were kept fixed to their single-crystal values. The refinement converged to good quality factors of *R*_p_ = 8.41% and *R*_wp_ = 8.36% with the following results for the quantification of the three phases: isoniazid saccharinate (A salt) 86.2(2)%, saccharin 8.2(2)%, and isoniazid 5.6(2)%. No further unassigned peaks were found in the difference pattern (Figure 5, lower panel).

### 2.3. DFT Structural Results

Fully periodic DFT calculations were carried out on both MH and A salt forms. Full optimization of the crystals’ structure, including both the unit-cell parameters and the atomic positions, was performed, the experimental SC-XRD structures of the salts being employed as starting geometry. Comparison of the optimized cell parameters and the key hydrogen-bond network descriptors for the two crystals with the corresponding experimental values (Table 2) shows overall good agreement. As expected, the calculations, which were performed at 0 K, yielded smaller unit cells and larger densities compared to those observed experimentally for both cocrystals, since no temperature effects were taken into account.

The periodic calculations yielded band gaps of 4.064 eV for the monohydrated crystal and 3.433 eV for the anhydrous phase, being within the expected values for organic salts containing aromatic rings and extensive hydrogen bonding (3.8–5.5 eV), and thereby classifying both phases as wide-band-gap organic materials. Although hybrid DFT band gaps may slightly underestimate the corresponding experimental optical gaps, the observed increase of approximately 0.63 eV upon hydration clearly indicates a significant influence of the water molecule on the electronic structure. The enlarged band gap in the monohydrate reflects greater localization of the electronic states and reduced charge-transfer interactions between the isoniazid and saccharinate ions, while the smaller gap of the anhydrous phase suggests enhanced electronic communication between the ionic components, leading to increased polarizability and optical activity.

**Figure 6:**
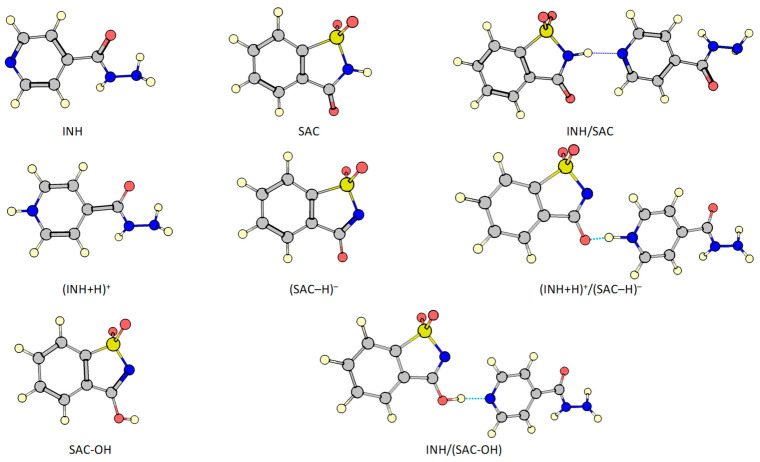
DFT/B3LYP/6-311++G(d,p)-optimized structures for the studied INH/SAC-based systems. The (INH+H)^+^/(SAC–H)^−^ structure is not a minimum (see text and Figure S1).

DFT calculations were also performed on the isolated INH and SAC molecules (and also for the hydroxy tautomer of saccharin, SAC-OH) and on different isolated (isoniazid/saccharin)-based dimeric structures, specifically: INH/SAC, (INH+H)^+^/(SAC–H)^−^ (which is present in the studied crystalline salts) and INH/(SAC-OH) (Figure 6). At the level of theory used in the calculations, the (INH+H)^+^/(SAC–H)^−^ isolated species was shown not to correspond to a minimum energy structure, the calculations converging to INH/(SAC-OH), with the proton initially located at the pyridine ring nitrogen of the isoniazid cation being transferred to the carbonyl oxygen atom of the saccharinate anion during optimization of the structure. The optimized calculated geometries for all calculated minimum energy species are given in the Supplementary Materials (Tables S17–S23).

The striking result of these calculations is the fact that the (INH+H)^+^/(SAC–H)^−^ isolated dimeric species does not correspond to a minimum energy structure, demonstrating its stabilization through intermolecular interactions in the crystalline state. As shown in Figure S1, during the optimization the (INH+H)^+^/(SAC–H)^−^ ionic dimeric unit converts into the molecular INH/(SAC-OH), which is ca. 17 kJ mol^−1^ more stable than the putative ionic dimer in gas phase.

The relative energies (zero-point-corrected) of the different isolated species, as predicted by the DFT/B3LYP/6-311++G(d,p) calculations, can be graphically seen in Figure S2. The tautomeric hydroxy form of saccharin (SAC-OH) is 35 kJ mol^−1^ higher in energy than the oxo tautomer. The INH/SAC dimer, established between the neutral molecules, is 16 kJ mol^−1^ more stable than the separate molecules, while the energy of the INH/(SAC-OH) dimer is 21 kJ mol^−1^ more stable than the isolated INH and SAC molecules (56 kJ mol^−1^ more stable than the isolated INH and (SAC-OH) species), and the (INH+H)^+^/(SAC–H)^−^ putative ionic dimeric unit stays ca. 4 kJ mol^−1^ below the INH and SAC isolated molecules and 424 kJ mol^−1^ below the separate ions.

### 2.4. Hirshfeld Surface Analysis

To analyze in detail interactions determining the packing characteristics of the INH-SAC salts, Hirshfeld surface analysis was undertaken, and the obtained information was compared with that concerning the crystals of pure INH (polymorph 1) [24,44] (CCDC 847197) and SAC (CCDC 274592) [34,45].

**Figure 7:**
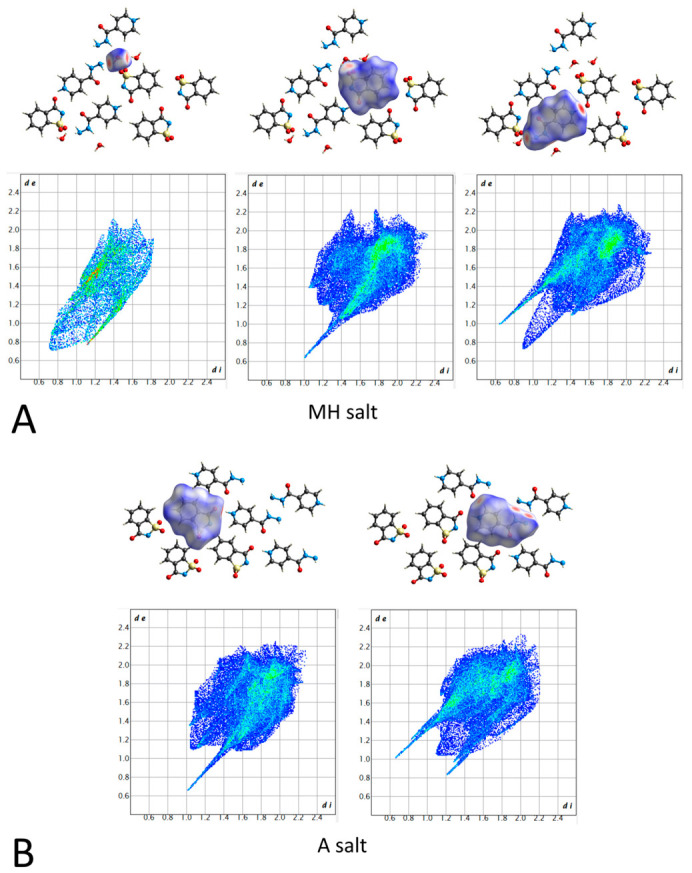
Hirshfeld surfaces and 2D fingerprint plots (all contacts) for the INH-SAC MH (plot (**A**)) and A (plot (**B**)) salts.

Maps of the normalized contact distances (*d*_norm_) to the Hirshfeld surfaces were obtained for the studied salts, together with the 2D fingerprint plots, in order to identify and quantify the different intermolecular contacts (Figure 7 and Figure S3–S7). The contributions of the different types of contact to the Hirshfeld surface maps for all crystals are provided in Table 3.

The maps of *d*_norm_ on the HS of the different molecules present in the two salts exhibit the characteristic bright red regions (short contacts) associated with the different N–H···O hydrogen-bond interactions, while the short contacts associated with the O_w_–H···O hydrogen bonds (subscript w indicates that the oxygen atom belongs to the water molecule) are also clearly noticeable in the case of the MH salt. H···O/O···H contacts (where the first atom is that inside the HS and the second is the one outside) account for over 30% and 40% of the *d*_norm_ mapping on the Hirshfeld surfaces of the INH and SAC units in both MH and A, respectively (Table 3), highlighting the relevance of the hydrogen-bonding networks in the two crystals. The cusp observed in the 2D fingerprint plots of the saccharinate ion (Figure 7) corresponds to the stronger N–H_(INH+H)_^+^···O(=C)_(SAC–H_^+^_)_^−^ hydrogen bond established between the protonated pyridine ring of the isoniazid cation and the carbonyl oxygen of the saccharinate (see also Figures S4 and S6). This is by far the closest contact involving the saccharinate in crystals of both MH and A salts, and has its complementary counterpart in the major cusp observed in the 2D fingerprint plots of the (INH+H)^+^ cation of the salts (see also Figures S3 and S5). N···H contacts account for ca. 10% of the HS in the salts and their individual organic components and are responsible for the additional, less pronounced cusps in the 2D fingerprint plots of the INH component of the crystals. The white regions of the HS are mostly related through π-π stacking interactions (flat regions) and contacts involving the C–H moieties of the aromatic rings of the two organic components. As a whole, C···C, N···N, C···N/N···C, and C···H/H···C contacts, which are representative of this type of interaction, account to ca. 18% of the HS surface area of the two salts. The C···O/O···C contacts, accounting for slightly less than 5% of the HS, are mostly associated with through-space bond–dipole interactions involving the carbonyl groups of the two organic components of the salts. On the other hand, H···H contacts represent dispersive interactions and constitute the second major type of contacts mapped on the HS, amounting to ca. 32% and 27% of the HS of MH and A, respectively (see Table 3). Some of the H···H contacts are rather short in the case of the MH salt, these being associated with water molecules.

**Figure 8:**
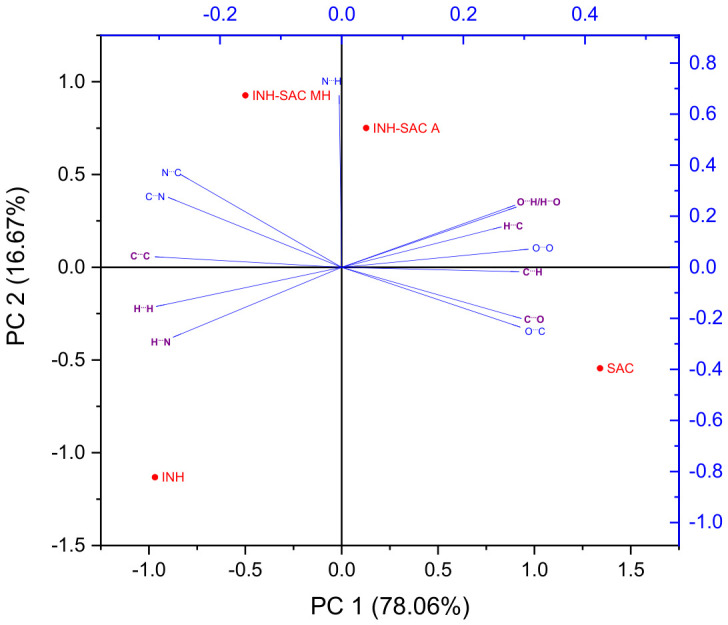
Score/loading PCA plot for the contact profiles for the INH-SAC MH and A salts, INH (polymorph 1) and SAC, based on the Hirshfeld surface maps of *d*_norm_. PCA was done using Origin OriginPro 2021 [51]. The data matrix was built with the % values for the different contacts given in Table 3 (N^…^N and N^…^O/O^…^N contacts excluded). The top and right axes correspond to loadings and the values for PC1 and PC2 are indicated for each contact type. The violet labels relate with the variables (contacts) that have larger values (more important contacts). The red dots correspond to the PC1 × PC2 scores for the different samples (crystals) defined according to the left and bottom axes.

According to a comparison of the percent distribution of the different contacts in the two salts, H···H contacts are somewhat more relevant in the monohydrated form than in the anhydrous salt (31.5% vs. 26.8%), while the opposite is observed for the C···H/H···C contacts (6.7% vs. 9.4%), but the general pattern of % distribution of the contacts in the two salts (and in the INH and SAC individual components in the two salts) is very similar. This similarity can also be clearly noticed in the graphical comparison of the profile of contacts in the two salts and those characteristic of the crystals of the pure components shown in Figure 8. The figure shows the results of Principal Component Analysis (PCA) [46,47,48,49,50] carried out using, as data, the % values for the different contacts obtained from the HS analysis presented in Table 3. In the plot, the top and right axes correspond to loadings and the values for PC1 and PC2 are indicated for each contact type (the violet labels relate with the variables (contacts) that have larger values—more important contacts). The red dots correspond to the PC1×PC2 scores for the different samples (crystals) defined according to the left and bottom axes. We can see the clear discrimination between the INH and SAC crystals along PC1 (which accounts for over 78% of variance in the data), with the first appearing on the left side of the shown score plot and the second on the right side, while the two salts occupy an intermediate position along PC1. On the other hand, PC2 discriminates the two ionic crystals (which appear for positive PC2 score values) from the two molecular crystals of the pure components, which exhibit negative PC2 scores. The PCA plot shown in Figure 8 is also very explicative regarding the major types of contacts determining the relative scores of the four crystals along PC1 and PC2. For example, along PC1, nitrogen-related contacts stay on the left side of the plot, the same side the nitrogen-rich INH stays on in the score plot, while oxygen-related contacts stay on the right side of the plot, same as the oxygen-richer SAC. As it could then be expected, the salts appear in the mid-region of the graph along PC1.

### 2.5. DSC, TGA and Hot-Stage Microscopy (HSM) Results

DSC and TGA analyses were performed to investigate the thermal behavior of the INH-SAC salts. The DSC traces of the pure components were also obtained (Figure S8) and were found to match those in the literature for polymorph **1** of INH and saccharin [24,34,52,53,54,55,56]. For INH a sharp endothermic melting peak at a *T*_m_ (onset) of 170.1 ± 0.5 °C, with Δ*H*_fus_ of 29.5 ± 0.5 kJ mol^−1^, was observed, while for saccharin, the melting peak was observed at a *T*_m_ (onset) of 227.3 ± 0.5 °C and Δ*H*_fus_ of 21.2 ± 0.5 kJ mol^−1^. The 1:1 physical mixture of the two materials exhibits a broad endothermic feature commencing significantly earlier, around 120 °C, which is characteristic of a simple physical mixture wherein the constituents initiate interaction or solubilization with each other upon heating, signifying that no stable cocrystal lattice was established in that particular sample.

**Figure 9:**
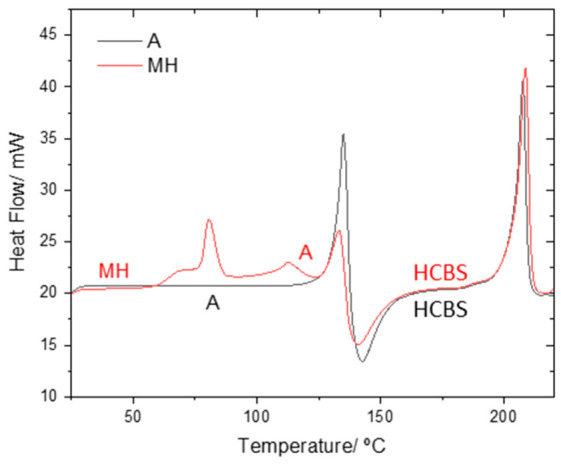
DSC curves (endothermic features pointing up) of the INH–SAC MH and A salt forms. *m*_MH_ = 5.60 mg; *m*_A1_ = 5.44 mg. β = 10 °C min^−1^.

**Figure 10:**
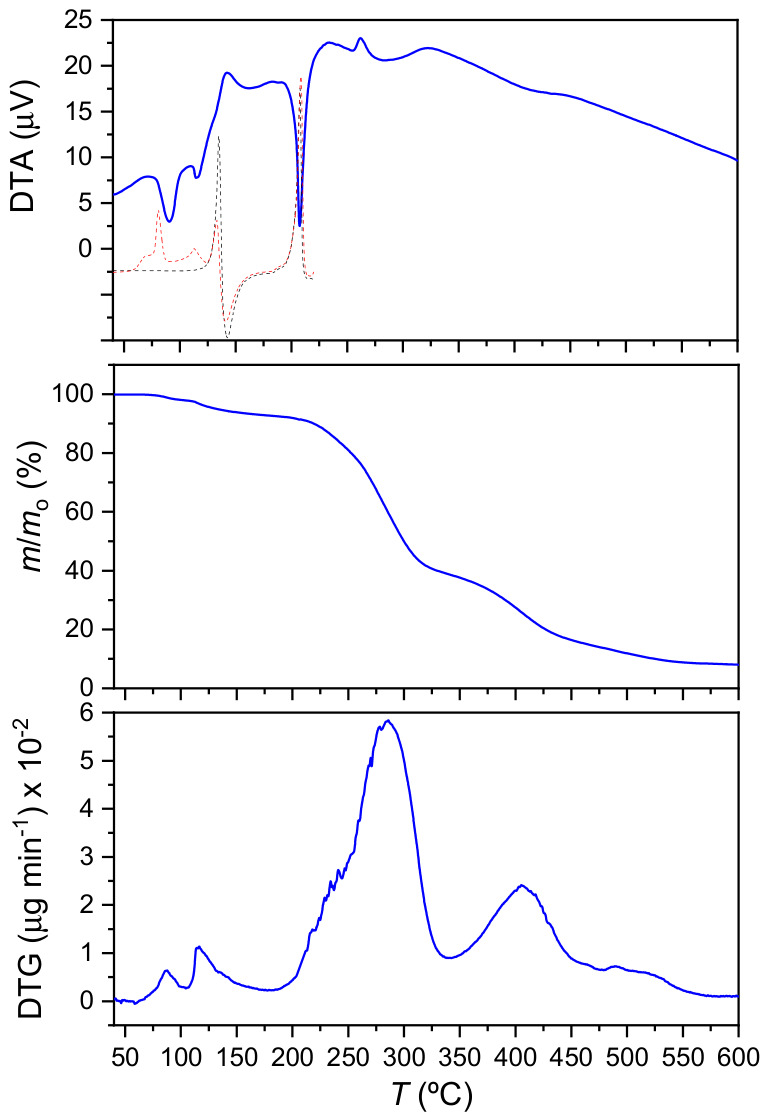
TG/DTA data for a sample of INH-SAC MH salt (initial mass, *m*_o_ = 7.63 mg). The DTA curve (**top** graph, blue line) shows the endothermic features pointing down; the dashed lines correspond to the DSC curves shown in Figure 9 (black curve for A and red curve for MH samples), shown for comparison. (**Middle**) and (**bottom**) panels present the TG and the derivative TG (d*m*/dt; DTG) curves.

The DSC of the anhydrous salt (Figure 9) shows a narrow endothermic peak at a *T*_m_ (onset) of 131.7 ± 0.5 °C and Δ*H*_fus_ of 25.0 ± 0.5 kJ mol^−1^, corresponding to the melting, which is followed by an exothermic peak with a maximum at 142.6 ± 0.5 °C, associated with formation of 2-[2-(pyridine-4-carbonyl)hydrazine-1-carbonyl]benzene-1-sulfonamide (HCBS), the INH-SAC adduct resulting from the nucleophilic acyl substitution at the saccharin carbonyl reaction leading to saccharin ring opening and formation of the new covalent hydrazide–amide derivative (see Section 2.6 on this subject). This material then melts at a *T*_m_ (onset) of 204.4 ± 0.5 °C (Δ*H*_fus_ of 43.5 ± 0.5 kJ mol^−1^). According to the performed thermogravimetry (TG/DTA) studies (Figure 10), the melting of HCBS is immediately followed by decomposition that takes place in four major steps (the first accompanying the melting) and is concluded at ca. 575 °C. In the first decomposition step, simultaneous with the melting of HCBS, ca. 5% of mass is lost, which fits well with the % mass of NH_3_ in the sample (5.0%), while ca. 50% of the mass is lost in the second (and most pronounced) step of decomposition, corresponding most probably to the release of SO_2_ and HNCO (×2). The remaining decomposition steps should then result from fragmentation of the most robust aromatic rings of the compound.

The DSC and TG/DTA traces of the monohydrate (MH) (see Figure 9 and Figure 10) are, as expected, more complex. Water release takes place in two closely spaced steps, corresponding to the endothermic broad peaks at ca. 74.8 and 108.0 °C. Dehydration results in partial formation of the anhydrous salt, that then melts, as testified by observation of the endothermic peak at *T*_m_ (onset) ~ 128.0 °C. Subsequently, formation of HCBS takes place, followed by the melting of this compound, as also observed in the DSC experiments where the starting material was the anhydrous salt form. The TG curve shows that until *T* ~ 95.0 and 120.0 °C, mass loss steps of ca. 2.5% (ca. 5% in total) take place, which fits well with the expected mass loss resulting from water release (5.3%), while, in consonance with the DSC results, only after the melting of HCBS are further mass losses observed, as described above.

It is worth noting that the (INH+H)^+^/(SAC–H)^−^ anhydrous salt exhibits a considerably lower melting point than both pure INH and SAC, in spite of the higher estimated sublimation enthalpy. This is a common observation for organic proton-transfer systems, built from large and flexible ions, which lead to a weaker packing efficiency and make the crystal lattice mechanically soft and thus easier to disrupt thermally. HCBS displays an intermediate melting point (higher than that of isoniazid but lower than that of saccharin).

**Figure 11:**
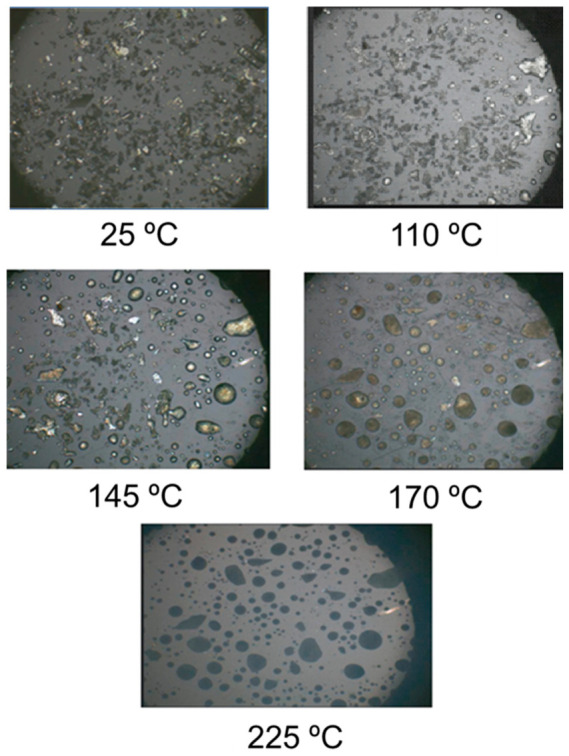
Hot-stage microscopy results (10× objective) during the heating of a sample of INH-SAC MH salt.

Results from hot-stage microscopy (HSM) on a sample of MH (Figure 11) further confirm the DSC and TG/DTA results. At the initial temperature of 25 °C, the sample comprises well-defined crystalline yellow particles. Upon a temperature increase up to 110 °C, these crystalline structures change their form visibly and lose their yellow color, which is consistent with the MH→A conversion, accompanied by the start of the melting. At 145 °C, melted material is dominant, and by 170 °C crystallization of HCBS has already taken place, the newly formed material taking the form of microcrystalline yellowish material. By 225 °C the melting of HCBS and sample decomposition have already taken place, and the material gains a dark color.

### 2.6. Infrared and Raman Spectroscopy

**Figure 12:**
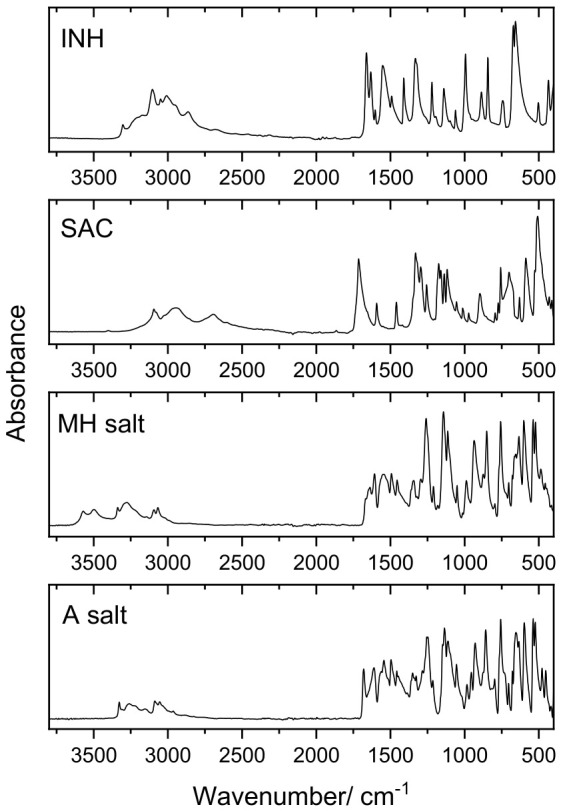
Infrared spectra of crystalline pure INH (polymorph 1) and SAC, and of their anhydrous (A) and monohydrated (MH) salts, at room temperature.

**Figure 13:**
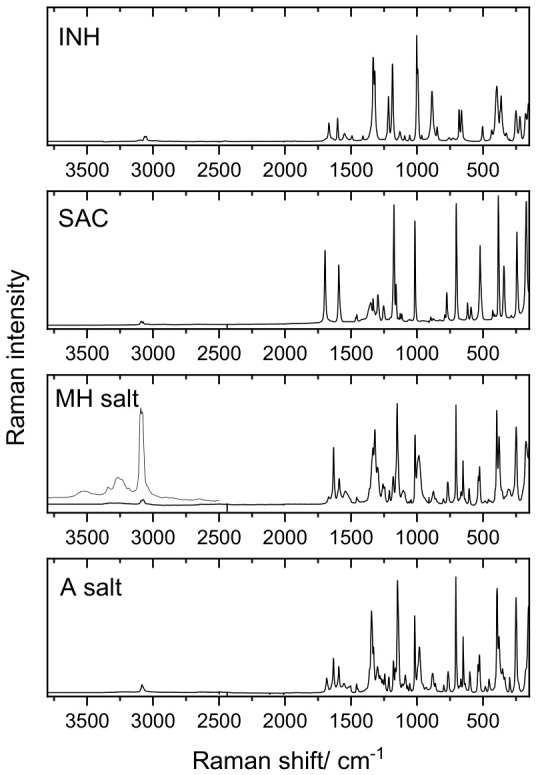
Raman spectra of crystalline pure INH (polymorph 1) and SAC, and of their anhydrous (A) and monohydrated (MH) salts, at room temperature.

The infrared and Raman spectra of the INH-SAC MH and A salts are shown in Figure 12 and Figure 13, respectively, together with those of the crystals of INH (polymorph **1**) and SAC. The spectra of the salts are also compared with those calculated at the DFT/B3LYP/6-31G(d,p)-D3 level of approximation (fully periodic) in Figures S9 and S10. Proposed assignments are provided in Tables S24 and S25. The description of the spectra of the two investigated salts by the calculations is excellent, with mean absolute errors (MAEs) of 20.4 and 20.2 cm^−1^ (1.48% and 1.64%, respectively) for IR and Raman spectra in the case of the anhydrous salt, and 11.0 and 17.4 cm^−1^ (0.79% and 1.36%) for IR and Raman spectra of the monohydrated salt. In the calculation of the MAE values for the anhydrous salt, the deviations in wavenumbers were determined as the difference between the observed wavenumbers and the average of the calculated values for the analogous vibrations belonging to the two active symmetry species in each technique (i.e., A_g_ and B_g_ modes in the case of Raman, and A_u_ and B_u_ modes for IR active modes).

The spectra of the crystals of the pure components are identical to those reported before [24,34]. Those of the salts show substantial differences compared to those of the crystals of the pure components. They differ from each other mostly in the high-frequency region where the bands resulting from the stretching modes of the water molecule are observed, which are absent in the spectra of the anhydrous salt, as expected. In the FTIR spectrum of MH, these bands are seen at 3570 (antisymmetric mode) and 3498 (symmetric) cm^−1^ (Figure 12 and Table S25), which have counterparts in the Raman spectrum at 3552 and 3509 cm^−1^, respectively (Figure 13 and Table S25). According to the calculations, the water bending mode contributes significantly to the bands observed at 1638 and 1607 cm^−1^ in infrared (1637 and 1596 cm^−1^ in Raman), while the water rotational modes give rise to the bands observed at 702, 478 and 434 cm^−1^ in the IR spectrum. In the Raman spectrum these bands are seen at 711 and 461 (two modes) cm^−1^, respectively.

Other band marks of MH, originated in the INH molecule, are observed at 3337 (νNH_2_ as), 3275 (νNH_2_ s), 3223 (νNH), 2507 (νNH(^…^O=)), 1667 (νC=O), 1650 (νNH(^…^O=)), 1638 (δNH_2_), 1549 (δNH), 947 (wNH_2_), and 797 (γNH) cm^−1^ in the IR spectrum, with counterparts in the Raman spectrum at similar frequencies (see Table S25), while infrared SAC-related MH mark bands are observed at 1184 (νSO_2_ as), 1075 (νSO_2_ s), 881 (νNS), 601 (δSO_2_), 538 (γSO_2_) and 502 (wSO_2_) cm^−1^ (1184, 1076, 892, 596, 545 and 495 cm^−1^ in Raman). In the IR spectrum of the anhydrous salt, the bands ascribed to the same modes are seen at 3338 (νNH_2_ as), 3305 (νNH_2_ s), 3261/3228 (νNH), 2514 (νNH(^…^O=)), 1680 (νC=O and νNH(^…^O=)), 1646 (δNH_2_), 1611 (δNH), 928 (wNH_2_), and 735 (γNH) cm^−1^ (INH-related modes), and 1285 (νSO_2_ as), 1101 (νSO_2_ s), 955 (νNS), 598 (δSO_2_ and γSO_2_) and 525 (wSO_2_) cm^−1^ (SAC-related modes), with the Raman bands being observed at frequencies close to those of the corresponding infrared bands (see Table S24). The experimental frequencies of all these vibrations are in good general agreement with the calculated values, as seen in Tables S24 and S25.

**Figure 14:**
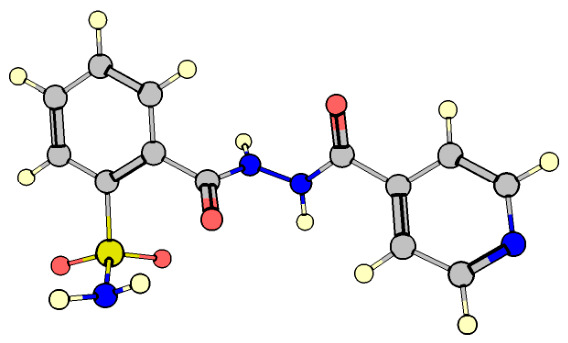
B3LYP/6-311++G(d,p)-optimized structure for HCBS. The input geometry from the calculations was extracted from the SC-XRD-solved crystalline phase.

**Figure 15:**
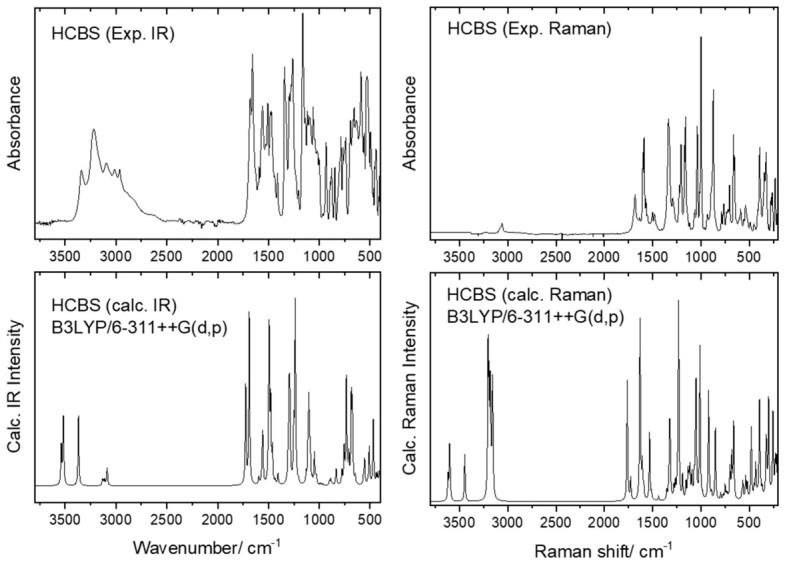
Experimental room temperature FTIR and Raman spectra of HCBS crystalline material and B3LYP/6-311++G(d,p)-calculated infrared and Raman spectra for the isolated molecule.

As indicated in Section 2.5, upon melting the INH-SAC anhydrous salt, the two compounds react to generate an adduct resulting from the nucleophilic acyl substitution at the saccharin carbonyl reaction, leading to saccharin ring opening and formation of the new covalent hydrazide–amide derivative, 2-[2-(pyridine-4-carbonyl)hydrazine-1-carbonyl] benzene-1-sulfonamide (HCBS) (Figure 14). Formation of this compound from isoniazid and saccharin has been reported before, and its crystal structure has been solved at room temperature by SC-XRD [57]. This reaction and the detailed structural and spectroscopic characterization of HCBS will be the object of a separate publication. B3LYP/6-311++G(d,p) calculations performed in the present study on the isolated molecule showed that HCBS is 27.4 kJ mol^−1^ less energetic than SAC and INH taken together (zero-point-corrected value; the optimized geometry of HCBS is provided in Table S26). Figure 15 shows the experimental FTIR and Raman spectra of crystalline HCBS as compared to the B3LYP/6-311++G(d,p)-calculated spectra for the monomeric isolated molecule, which agrees fairly well with the experimental data (see also Table S27, with assignments). We solved the structure of the produced crystalline HCBS by SC-XRD, our results being in perfect agreement with those reported before [57]; thus, the identification of this compound as the product of the reaction between INH and SAC upon melting of the anhydrous salt, as pointed out in Section 2.5, is doubtless.

## 3. Experimental and Computational Methods

### 3.1. Materials

Isoniazid (or isonicotinic acid hydrazide), 98% purity, was purchased from ABCR GmbH, Karlsruhe-Knielingen, Germany, and saccharin (>99%) was obtained from Sigma Aldrich, Darmstadt, Germany. The solvents used in the different synthesis procedures were spectroscopic-grade: methanol (Honeywell, Charlotte, NC, USA), acetone (Carlo Erba Reagents, Val-de-Reuil France), and acetonitrile (ISOLAB Laborgeräte GmbH, Eschau, Germany). Distilled and deionized water was used throughout the study.

### 3.2. Experimental Instrumentation and Procedures

Single-crystal X-ray diffraction (SC-XRD): The synthesized crystals were screened on a polarizing microscope (LEICA S8APO, Letzlar, Germany). Suitable crystals of MH and A salt forms were chosen for SC-XRD data collection, with approximate dimensions of 0.1 × 0.3 × 0.3 and 0.3 × 0.2 × 0.2 mm^3^, respectively. The crystals were mounted on a glass fiber and the data were acquired at room temperature in a Bruker D8 QUEST diffractometer (Bruker Coorporation, Billerica, MA, USA), using MoKα radiation (*λ* = 0.71073 Å). Data collection, cell refinement, and integration were performed using the Bruker APEX (version 6) software package [58], and the structures were solved using SHELXT-2018/2 [59], via the Intrinsic Phasing method, and refined with SHELXL-2018/3 [60], by full matrix least-squares minimization, as implemented in Olex2 (version 1.5) [61]. Non-hydrogen atoms were refined anisotropically, while hydrogen atoms were refined using SHELXL-2018/3 default riding values, except for those attached to N atoms, which, being involved in hydrogen bonding, had their coordinates freely refined with *U*_iso_ constrained to 1.2× those of their parent atoms. Crystallographic data and refinement parameters are summarized in Table 1. Structural details are given in the Supplementary Materials (X-Ray structural data section; Tables S1–S16). The CIF files containing the supplementary crystallographic data for the two salt forms were deposited at the Cambridge Crystallographic Data Centre (CCDC), with the reference numbers 2551514 (MH) and 2554377 (A).

Powder X-ray diffraction (PXRD): PXRD measurements were conducted at room temperature in a Rigaku Miniflex 600 (Tokyo, Japan), equipped with a D/Tex Ultra 1D detector or a Malvern PANalytical Empyrean Series 3 X-ray diffractometer (Malvern, UK). The ground powdered sample was placed on the sample chamber of the instrument (5 mm diameter and 0.1 mm deep), and the diffractograms were obtained in Bragg–Brentano geometry within the 2*θ* range of 3–40°, at a rate of 5° min^−1^, and using Cu Kα (1.541862 Å) radiation. Simulated PXRD patterns were generated from the single-crystal data using Mercury [62]. Analysis of the PXRD data was conducted via Rietveld refinements performed with *Profex* (version 5.6.1) [63]) using the structural parameters obtained from the single-crystal data.

Differential scanning calorimetry (DSC) and thermogravimetric analysis (TGA): DSC experiments were performed on a Perkin Elmer DSC 4000 instrument (Waltham, MA, USA), under a N_2_ atmosphere. Approximately 5 mg of each sample was sealed in an aluminum pan and heated from 25 °C to 280 °C at a rate of 10 °C min^−1^. Temperature and enthalpy calibrations were performed using caffeine (Mettler Toledo calibration substance, ME 18872, *T*_m_ = 235.6 ± 0.2 °C) and indium (Perkin Elmer (Waltham, MA, USA), 99.99%, *T*_m_ = 156.6 °C; Δ*H*_fus_ = 3286 ± 13 J mol^−1^). TGA measurements on the INH-SAC MH salt (~7.5 mg) were carried out on a Seiko Exstar TG/DTA 6200 instrument (Chiba, Japan), within the temperature range 20–600 °C. The sample was placed in a ceramic sample pan and the experiments were carried out under an inert (N_2_) atmosphere. A heating rate of 10 °C min^−1^ was used. Weighing technique precision was ±0.01 mg.

Hot-stage microscopy (HSM): For HSM experiments, a LEICA S8APO microscope (Letzlar, Germany) was used, with a 10× objective, together with a Linkam hot-stage model THMS350V and LNP96-S controller (Linkam Scientific Instruments, Tadworth, UK).

Infrared (IR) and Raman spectroscopy: IR spectra of the powdered materials were recorded in the attenuated total reflectance (ATR) mode, using a diamond-based accessory, in a Bruker Invenio spectrometer (Bruker Coorporation, Billerica, MA, USA), equipped with a deuterated triglycine sulphate (DTGS) detector and a KBr beamsplitter, in the range of 400–4000 cm^−1^, with 2 cm^−1^ resolution and 64 accumulations. During the experiments, the spectrometer optics and the sample compartment were purged by a stream of dry air to avoid interference of atmospheric water vapor. Raman spectra were collected within the 4000–4200 cm^−1^ Raman shift range in a Horiba Xplora^TM^ Plus micro-Raman system (Kyoto, Japan), in the backscattering geometry and confocal mode (0.5 μm XY), with 785 nm laser excitation. Spectral resolution was 10 cm^−1^, a 10× objective lens (numerical aperture 0.25) was used, and maximum output power, number of accumulations per spectrum and integration time were 50% (~50 mw at the sample), 5 and 1 s, respectively. Wavenumbers were calibrated using the silicon wafer band at 520.7 cm^−1^. Detection was achieved using a Peltier air-cooled (−60 °C) CCD detector (Horiba Scientific, 1024 × 256 pixels) with an edge filter to block Rayleigh scattering. In the temperature variation experiments, a Linkam hot-stage (model LTS420) was used together with an LNP96-S controller (Linkam Scientific Instruments, Tadworth, UK).

### 3.3. Density Functional Theory Calculations

DFT calculations were performed on the isolated INH/SAC-relevant systems using Gaussian 16 (Revision A.03) [64] using the Becke 3-parameters; Lee, Yang and Parr (B3LYP) functional [65,66,67]; and the extended 6-311++G(d,p) basis set [68,69,70]. Fully periodic DFT calculations on the MH and A salt forms were carried out using CRYSTAL17 [71], with the B3LYP functional, the 6-31G(d,p) [72,73] basis set (used instead of the 6-311++G(d,p) basis set due to limited computational resources), and the Grimme D3 dispersion correction [74]. Calculations used an extra-large numerical integration grid (99 and 974 radial and angular points), an SCF convergence criterion of 10^−8^ Hartree, k-point mesh 4 × 4 × 4, and the program standard geometry optimization criteria (energy change < 10^−6^ Hartree, gradient < 3 × 10^−4^ Hartree Bohr^−1^, and displacement < 1.2 × 10^−3^ Bohr). Full lattice optimization was carried out, and vibrational spectra (Raman and IR) were calculated at the same level of theory, wavenumbers being scaled by 0.95 and 0.970, above and below 1800 cm^−1^ [24,34] respectively.

### 3.4. Hirshfeld Surface Analysis

Hirshfeld surface analysis [35,36,37,38,39,40] was performed using Crystal Explorer 21 [75], based on the CIF file of the INH-SAC MH and A salt forms obtained from the present SC-XRD investigation. Maps of the normalized contact distances to the calculated Hirshfeld surfaces,

(1) $$d_{norm}=\frac{d_{i}-r_{i}^{vdW}}{r_{i}^{vdW}}+\frac{d_{e}-r_{e}^{vdW}}{r_{e}^{vdW}},$$

where *r_i_^vdW^* are the van der Waals radii and *d_e_* and *d_i_* represent the distances from a surface point to the nearest atom inside and outside the surface, respectively, were generated alongside the corresponding 2D fingerprint plots [40] to quantify the contributions of different intermolecular contacts.

## 4. Conclusions

Two new crystalline proton-transfer forms based on isoniazid and saccharin, namely the monohydrated salt MH, (INH+H)^+^/(SAC–H)^−^·H_2_O, and the anhydrous salt A, (INH+H)^+^/(SAC–H)^−^, were successfully synthesized and comprehensively characterized through SC-XRD, PXRD, DSC/TGA, hot-stage microscopy, infrared and Raman spectroscopy, Hirshfeld surface analysis, and DFT calculations. The obtained results demonstrate that the presence or absence of water during synthesis plays a decisive role in directing formation of the hydrate or anhydrous phase, respectively.

Single-crystal X-ray diffraction revealed that both salts are genuine proton-transfer systems in which protonation of the pyridine nitrogen atom of isoniazid and deprotonation of saccharin lead to robust ionic assemblies stabilized predominantly by strong N–H···O hydrogen bonds. PXRD and Rietveld analyses confirmed the bulk-phase purity of the hydrate and the predominance of the anhydrous salt in the corresponding samples.

The fully periodic DFT calculations reproduced satisfactorily the experimentally determined structural parameters and hydrogen-bond motifs, and were useful to help interpret the experimentally obtained vibrational data. Calculations on isolated molecular and ionic species further demonstrated that the ionic (INH+H)^+^/(SAC–H)^−^ arrangement is not intrinsically stable in the gas phase, converting spontaneously into the neutral INH/(SAC-OH) form during optimization. This result highlights the crucial role played by crystal packing and intermolecular interactions in stabilizing the proton-transfer structures in the solid state.

Hirshfeld surface analysis and PCA of the contact distributions evidenced the intermediate structural character of the salts relative to the parent INH and SAC crystals and emphasized the importance of nitrogen- and oxygen-related contacts in defining the packing features of these ionic solids.

Thermal studies demonstrated that the monohydrate undergoes dehydration upon heating, converting into the anhydrous form before melting. The anhydrous salt melts at relatively low temperature, *T*_m_ (onset) = 131.7 ± 0.5 °C, reflecting the softer packing typically found in molecular ionic solids composed of large flexible ions. Upon melting, a chemical transformation through nucleophilic acyl substitution at the saccharin carbonyl group, leading to ring opening and formation of the new covalent hydrazide–amide derivative HCBS, takes place. HCBS subsequently melts at 204.4 ± 0.5 °C before decomposing.

Infrared and Raman spectroscopic investigations, supported by DFT calculations, enabled complete identification of the characteristic vibrational signatures of both salts and clearly distinguished the hydrate and anhydrous phases, particularly through the presence or absence of water-related vibrational modes. The spectroscopic data further confirmed the proton-transfer nature of the crystalline materials and the structural changes associated with thermal conversion and HCBS formation.

Overall, the present work demonstrates that saccharin is an effective coformer for isoniazid, enabling formation of stable proton-transfer crystalline materials with distinct structural and thermal properties. Beyond their crystallographic and spectroscopic interest, these systems may offer potential advantages for pharmaceutical formulation, including improved organoleptic properties and modified solid-state behavior. In addition, the observed transformation into HCBS upon heating reveals an interesting reactive pathway between isoniazid and saccharin that deserves further investigation. The results reported here contribute to the growing understanding of API–excipient proton-transfer systems and provide new insights into the solid-state chemistry of isoniazid-based pharmaceutical materials.

## Figures and Tables

**Table 1:** Crystal and structural refinement data for MH [(INH+H)^+^/(SAC–H)^−^.H_2_O] and A [(INH+H)^+^/(SAC–H)^−^] salts.

	MH (INH+H)^+^/(SAC–H)^−^.H_2_O	A (INH+H)^+^/(SAC–H)^−^
Empirical formula	C_13_H_14_N_4_O_5_S	C_13_H_12_N_4_O_4_S
Formula weight	338.34	320.33
Temperature (K)	300(1)	298(1)
Crystal system	triclinic	monoclinic
Space group	*P*-1	*P*2_1_/*c*
*a* (Å)	7.3442(4)	11.3749(3)
*b* (Å)	8.0527(4)	7.9243(2)
*c* (Å)	12.5571(6)	15.3365(4)
α (°)	80.968(2)	90
β (°)	83.032(2)	98.636(2)
γ (°)	84.994(2)	90
Volume (Å^3^)	726.25(6)	1366.73(6)
*Z*, *Z′*	2/1	4/1
ρ_calc_ (g/cm^3^)	1.547	1.557
μ (mm^−1^)	0.256	0.263
*F*(000)	352.0	664
Crystal size (mm^3^)	0.3 × 0.3 × 0.1	0.3 × 0.2 × 0.2
Radiation	Mo Kα (λ = 0.71073 Å)	Mo Kα (λ = 0.71073 Å)
2*θ* range for data collection/°	5.602–50.826	5.374–49.992
Index ranges	−8 ≤ *h* ≤ 8, −9 ≤ *k* ≤ 9, −15 ≤ *l* ≤ 15	−13 ≤ *h* ≤ 13, −9 ≤ *k* ≤ 9, −18 ≤ *l* ≤ 18
Reflections collected	28,343	155,631
Independent reflections	2647 [*R*_int_ = 0.0285, *R*_sigma_ = 0.0123]	2407 [*R*_int_ = 0.1011, *R*_sigma_ = 0.0324]
Data/restraints/parameters	2647/4/232	2407/0/212
Goodness-of-fit on *F*^2^	1.090	1.120
Final *R* indexes [*I* >= 2σ (*I*)]	*R*_1_ = 0.0376, w*R*_2_ = 0.0909	*R*_1_ = 0.0470, w*R*_2_ = 0.0951
Final *R* indexes [all data]	*R*_1_ = 0.0426, w*R*_2_ = 0.0965	*R*_1_ = 0.0781, w*R*_2_ = 0.1090
Largest diff. peak/hole (e Å^−3^)	0.26/−0.31	0.22/−0.31

**Table 2:** DFT/B3LYP/6-31G(d,p) calculated structural data (fully periodic calculations) for the INH-SAC MH and A salts, as compared to the experimentally obtained values.

	MH			A	
	Exp.	Calc.		Exp.	Calc.
*a*/Å	7.3442(4)	6.9428		11.3749(3)	11.2469
*b*/Å	8.0527(4)	7.8011		7.9243(2)	7.7924
*c*/Å	12.5571(6)	12.6752		15.3365(4)	14.4696
*α*/°	80.968(2)	77.449		90	90
*β*/°	83.032(2)	81.433		98.636(2)	98.978
*γ*/°	84.994(2)	81.012		90	90
*V*/Å^3^	726.24	657.15		1366.73(6)	1268.13
Band gap/eV		4.064			3.433
(H^…^A, D^…^A/Å, angle/°)					
N2–H2^…^O1	1.68(2), 2.642(2), 177(2)	1.589, 2.651, 174.7	N2–H2^…^O1	1.69(3), 2.656(2), 174(2)	1.562, 2.606, 172.8
N3–H3^…^O5 *^a^*	2.04(3), 2.859(3), 159(2)	1.739, 2.727, 160.3	N3–H3^…^N3 *^h^*	2.60(3), 3.149(4), 121(2)	2.330, 2.500, 117.0
N4–H4A^…^O2 *^b^*	2.52(3), 3.056(3), 122(3)	2.318, 2.937, 118.0	N3–H3^…^N4 *^h^*	2.11(3), 2.925(3), 153(2)	1.862, 2.815, 152.5
N4–H4A^…^O4	2.31(3), 2.733(3), 111(2)	2.374, 2.753, 100.8	N4–H4A^…^O3 *^i^*	2.45(3), 3.169(3), 136(2)	2.200, 3.110, 143.6
N4–H4B^…^O5 *^c^*	2.40(3), 3.136(3), 144(3)	2.126, 2.997, 141.8	N4–H4B^…^O3 *^j^*	2.43(3), 3.042(3), 130(2)	2.178, 2.910, 122.4
O5–H5A^…^O2 *^d^*	2.59(4), 3.289(3), 133(3)	*^m^*	C8–H8^…^O4 *^k^*	2.39, 3.217(3), 147.5	2.123, 3.079, 146.4
O5–H5A^…^O3	2.23(4), 2.831(2), 122(2)	*^m^*	C2–H2A^…^O2 *^l^*	2.50, 3.311(3), 145.8	2.144, 3.088, 145.4
O5–H5B^…^O5 *^e^*	2.04(5), 2.934(4), 141(4)	*^m^*			
C2–H2A^…^O2 *^d^*	2.60, 3.492(2), 160.9	2.648, 3.598, 146.3			
C8–H8^…^O4 *^f^*	2.63, 3.439(3), 145.2	2.324, 3.274, 145.3			
C9–H9^…^O2 *^g^*	2.64, 3.313(3), 129.5	2.475, 3.442, 148.4			
C12–H12^…^N1	2.69, 3.354(2), 128.8	2.475, 3.312, 133.3			

*^a^* −1 + *x*, *y*, 1 + *z*; *^b^* −*x*, 2 − *y*, 1 − *z*; *^c^* −*x*, 2 − *y*, 1 − *z*; *^d^* 1 − *x*, 1 − *y*, −*z*; *^e^* 2 − *x*, 1 − *y*, −*z*; *^f^* *x*, −1 + *y*, *z*; *^g^* −*x*, 1 − *y*, −*z*; *^h^* −*x*, 2 − *y*, 1 − *z*; *^i^* −1 + *x*, 1 + *y*, *z*; *^j^* 1 − *x*, 2 − *y*, 1 − *z*; *^k^* *x*, 1 − *y*, *z*; *^l^* 2 − *x*, −½ + *y*, ½ − *z*. *^m^* The calculated values for the H bonds where water appears as a donor are not directly comparable with the experimental ones because the calculations did not take into account the water disorder observed experimentally.

**Table 3:** Partitioning (%) of the Hirshfeld surface area of the INH-SAC MH and A salts by the different types of intermolecular contacts, compared to those of pure crystals of INH (polymorph 1) and SAC *^a^.*

Total Area/Å^2^	MH: INH Unit 167.4	MH: SAC Unit 178.2	MH: H_2_O Unit 42.1	MH 387.7	A: INH Unit 168.9	A: SAC Unit 180.5	A 349.4	INH Polymorph 1 [24,44] 165.3	SAC [34,45] 183.28
C/C	8.8	8.3		**7.6**	7.5	7.1	**7.3**	8.8	4.0
C/H	8.8	6.3		**6.7**	10.3	9.4	**9.9**	6.6	10.5
C/N	3.6	3.5		**3.1**	2.3	2.0	**2.2**	2.5	0.6
C/O	5.0	3.0		**3.5**	5.5	4.3	**4.9**	3.7	12.3
N/N	0.6	0.4		**0.4**	0.0	0.0	**0.0**	0.1	0.0
N/H	10.6	11.3		**9.8**	10.8	10.1	**10.5**	20.9	2.9
N/O	0.1	0.0		**0.0**	0.7	0.3	**0.5**	0.0	0.0
O/O	0.6	0.4	0.0	**0.4**	1.0	1.2	**1.1**	0.1	1.6
O/H	30.7	42.5	36.7	**36.8**	31.8	41.6	**36.8**	14.3	52.8
H/H	31.2	24.4	63.3	**31.5**	30.1	23.7	**26.8**	42.9	15.5

*^a^* Percentages refer to reciprocal (inside/outside and outside/inside the Hirshfeld surface) contacts. In all crystals, intermolecular contacts involving the S atom are calculated as not contributing to the *d*_norm_ map on the Hirshfeld surface. For the salts, the percentages were obtained from the surface-weighted calculated percentages for the different species present in the asymmetric unit.

## Data Availability

The data supporting this article have been included as part of the Supplementary Materials. CIF files for the MH and A INH-SAC salts have been deposited in CCDC, Nº 2551514 and 2554377, respectively.

## Supplementary Materials

The following supporting information can be downloaded at: https://www.mdpi.com/article/10.3390/molecules31122187/s1, X-ray structural data section: data for INH-SAC mono-hydrated (MH) salt crystal (Tables S1–S8); data for INH-SAC anhydrous salt crystal (Tables S9–S16); Cartesian coordinates of the optimized isolated species (Tables S17–S23 and S26); tables with vibrational assignments for A, MH and HCBS (Tables S24, S25 and S27); Figure S1: Change in energy during geometry optimization for the (INH+H)+/(SAC–H)^−^ isolated dimer; Figure S2: Relative energies of the different INH and SAC isolated systems investigated in this study; Figures S3–S7: The calculated Hirshfeld surfaces and 2D fingerprint plots for the different contacts for the different systems; Figure S8: The DSC curves of pure INH (polymorph 1) and SAC; Figures S9 and S10: The comparison of the experimental IR and Raman spectra of A and MH with the DFT-calculated (periodic) spectra.

## References

[1] Saini V., Goyal A., Kumar A. (2022). Isoniazid: An Exploratory Review. Asian J. Org. Med. Chem..

[2] Rishanlang N., Rohit B., Ridahunlang N. (2023). Isoniazid Derivatives as Anti-tubercular Agents: From Structural Design to Clinical Investigations. Infect. Disord. Drug Targets.

[3] Bhat S.I. (2025). With a Word of Caution in Geriatric Population. Ann. Gerontol. Geriatr. Res..

[4] Shakya N., Garg G., Agrawal B., Kumar R.K. (2012). Chemotherapeutic Interventions Against Tuberculosis. Pharmaceuticals.

[5] Nair V., Okello M., Mangu N.K., Seo B.I., Gund M. (2015). A Novel Molecule with Notable Activity Against Multi-Drug Resistant Tuberculosis. Bioorg. Med. Chem. Lett..

[6] Singh R., Kumar P., Tahlan K. (2020). Drugs Against Mycobacterium Tuberculosis, in Drug Discovery Targeting Drug-Resistant Bacteria.

[7] Stagg H.R., Lipman M., McHugh T.D., Jenkins H.E. (2017). Isoniazid-Resistant Tuberculosis: A Cause for Concern?. Int. J. Tuberc. Lung Dis..

[8] Zhou L., Liu E., Lu Y., Wang L., Duanmu H., Chanyasulkit C., Strong A.J., Zhang H. (2017). Preventive Therapy Against Tuberculosis. Handbook of Global Tuberculosis Control.

[9] Vojnov L., Venter W.D.F. (2022). Isoniazid Prophylaxis: Highly Effective But Underutilised to Prevent Tuberculosis in People Living with HIV. Lancet Glob. Health.

[10] Laux T.S., Walker A., Agarwal A., Jain Y. (2023). Isoniazid for Preventing TB in HIV-Positive People in Infectious Diseases and Neglected Tropical Diseases.

[11] Winder F.G., Collins P.B. (1970). Inhibition by Isoniazid of Synthesis of Mycolic Acids in Mycobacterium tuberculosis. Microbiology.

[12] Schroeder E.K., Souza O.N., Santos D.S., Blanchard J.S., Basso L.A. (2002). Drugs that Inhibit Mycolic Acid Biosynthesis in Mycobacterium tuberculosis. Curr. Pharm. Biotech..

[13] Vilchèze C. (2020). Mycobacterial Cell Wall: A Source of Successful Targets for Old and New Drugs. Appl. Sci..

[14] Collins D.M., Bowen D.A.L. (1953). Isoniazid in the Treatment of Pulmonary Tuberculosis. Tubercle.

[15] Milanov V., Yanev N., Gabrovska N., Dimitrova D., Bachiyska E., Youroukova V. (2022). Current Approaches to Control of Isoniazid-Resistant Tuberculosis. Probl. Infect. Parasit. Dis..

[16] Yandhi R., Ahmad Z. (2024). Management of Isoniazid Monoresistant Tuberculosis. Biosci. Med..

[17] Bhutani H., Sing S., Jindal K.C. (2005). Drug-Drug Interaction Studies on First-Line Anti Tuberculosis Drugs. Pharm. Dev. Technol..

[18] Haywood A., Mangan M., Grant G., Glass B. (2005). Extemporaneous Isoniazid Mixture: Stability Implications. J. Pharm. Pract. Res..

[19] Bhutani H., Singh S., Jindal K.C., Chakraborti A.K. (2005). Mechanistic Explanation to the Catalysis by Pyrazinamide and Ethambutol of Reaction Between Rifampicin and Isoniazid in Anti-TB FDCs. J. Pharm. Biomed. Anal..

[20] Sankar R., Sharda N., Singh S. (2003). Behavior of Decomposition of Rifampicin in the Presence of Isoniazid in the pH Range 1–3. Drug Dev. Ind. Pharm..

[21] Bhutani H., Mariappan T.T., Singh S. (2004). An Explanation for the Physical Instability of a Marketed Fixed Dose Combination (FDC) Formulation Containing Isoniazid and Ethambutol and Proposed Solutions. Drug Dev. Ind. Pharm..

[22] Mashhadi S.M.A., Batsanov A.S., Sajjad S.A., Nazir Y., Bhatti M.H., Yunus U. (2021). Isoniazid-Gentisic Acid Cocrystallization: Solubility, Stability, Dissolution Rate, Antioxidant and Flowability Properties Studies. J. Mol. Struct..

[23] Salem A., Khanfar E., Nagy S., Széchenyi A. (2022). Cocrystals of Tuberculosis Antibiotics: Challenges and Missed Opportunities. Int. J. Pharm. Sci..

[24] Inan N.I., Syeitkhajy A., Yilmaz A., Sarier N., Ildiz G.O., Fausto R. (2026). Synthesis, Structure, Vibrational Spectra and Thermal Analysis of Isoniazid-Succinic Acid Cocrystal. Photochem. Spectrosc..

[25] Majewski M., Chruścicka I., Buchta J., Egierska D., Burzyńska P., Pietruszka P., Perszke M., Całkosiński A. (2020). What Do We Know About Sugar Substitutes. J. Educ. Health Sport.

[26] Kharat S., Mali S. (2024). Navigating Weight Management with Stevia: Insights into Glycemic Control. New Insights Obes. Gene. Beyond..

[27] Begum R.F., Nirenjen S., Rushendran R., Manisha M., Pavithra N., Sridevi S., Singh S.A. (2025). Exploring the Impact of Artificial Sweeteners on Diabetes Management and Glycemic Control. Front. Nutr..

[28] Imanto T., Wikantyasning E.R., Nurwaini S., Amalia M., Sambudi N.S., Harun N.Y. (2024). Preparation and Solid-State Characterization of Ketoprofen-Succinic Acid-Saccharin Cocrystal with Improved Solubility. Int. J. Appl. Pharm..

[29] Sopyan I., Layyareza R.T., Megantara S., Marvita S.S. (2023). Carvedilol Solubility Enhancement by Multicomponent Crystallization with Coformers of Benzoic Acid, Isonicotinamide, and Saccharin. Pharmacia.

[30] Butchko H.H., Stargel W.W., Comer C.P., Mayhew D.A., Benninger C., Blackburn G.L., Sonneville L.M.J., Geha R.S., Hertelendy Z., Koestner A. (2002). Aspartame: Review of Safety. Regul. Toxicol. Pharmacol..

[31] Qurrat-ul-Ain, Khan S. (2015). Artificial Sweeteners: Safe or Unsafe?. J. Pak. Med. Assoc..

[32] Patel C., Kumaresan S. (2022). Artificial Sweeteners: A Review. Int. J. Community Dent..

[33] Sawma M.J., Zayyat R.M., Ayoub G.M., Mahmassani N. (2025). Environmental and Health Impacts of Selected Artificial Sweeteners: Effectiveness of Treatment Methods and Innovative Approaches for Mitigation. Results Chem..

[34] Syeitkhajy A., Yilmaz A., Sarier N., Ildiz G.O., Fausto R. (2026). Picolinamide-Saccharin Cocrystal: Synthesis, Structure, Vibrational Spectra and Thermal Behavior. J. Mol. Struct..

[35] Hirshfeld F.L. (1977). Bonded-Atom Fragments for Describing Molecular Charge Densities. Theor. Chim. Acta.

[36] Spackman M.A., Byrom P.G. (1977). A Novel Definition of a Molecule in a Crystal. Chem. Phys. Lett..

[37] McKinnon J.J., Spackman M.A., Mitchell A.S. (2004). Novel Tools for Visualizing and Exploring Intermolecular Interactions in Molecular Crystals. Acta Cryst. B.

[38] Spackman M.A., Jayatilaka D. (2009). Hirshfeld Surface Analysis. CrystEngComm.

[39] McKinnon J.J., Jayatilaka D., Spackman M.A. (2007). Towards Quantitative Analysis of Intermolecular Interactions with Hirshfeld Surfaces. Chem. Commun..

[40] Spackman M.A., McKinnon J.J. (2002). Fingerprinting Intermolecular Interactions in Molecular Crystals. CrystEngComm.

[41] Akalin E., Akyuz S. (2007). Vibrational structure of Free and Hydrogen Bonded Complexes of Isoniazid: FT-IR, FT-Raman and DFT Study. J. Mol. Struct..

[42] Borba A., Gómez-Zavaglia A., Fausto R. (2009). Molecular Structure, Infrared Spectra, and Photochemistry of Isoniazid under Cryogenic Conditions. J. Phys. Chem. A.

[43] Lemmerer A., Bernstein J., Kahlenberg V. (2010). One-pot Covalent and Supramolecular Synthesis of Pharmaceutical Co-Crystals Using the API Isoniazid: A Potential Supramolecular Reagent. CrystEngComm.

[44] Lemmerer A. (2012). Covalent Assistance to Supramolecular Synthesis: Modifying the Drug Functionality of the Antituberculosis API Isoniazid in situ During Co-Crystallization with GRAS and API Compounds. CrystEngComm.

[45] Wardell J.L., Low J.N., Glidewell C. (2005). Saccharin, Redetermined at 120 K: A Three-Dimensional Hydrogen-Bonded Framework. Acta Cryst. E.

[46] Pearson K. (1901). On Lines and Planes of Closest Fit to Systems of Points in Space. Phil. Mag..

[47] Hotelling H. (1933). Analysis of a Complex of Statistical Variables into Principal Components. J. Educ. Psychol..

[48] Hotelling H. (1933). Analysis of a Complex of Statistical Variables into Principal Components. J. Educ. Psychol..

[49] Hotelling H. (1936). Relations Between Two Sets of Variates. Biometrika.

[50] Ildiz G.O., Bayari S.H., Yorguner N., Fausto R., El-Baz A.S., Mahmoud A. (2021). Blood Serum Infrared Spectra Based Chemometric Models for Auxiliary Diagnosis of Autism Spectrum Disorder. Neural Engineer Techniques for Autism Spectrum Disorder.

[51] (2021). OriginPro.

[52] Ngilirabanga J.B., Aucamp M., Pires Rosa P., Samsodien H. (2020). Mechanochemical Synthesis and Physicochemical Characterization of Isoniazid and Pyrazinamide Co-crystals with Glutaric Acid. Front. Chem..

[53] Fernandes G.F.S., Salgado H.R.N., dos Santos J.L. (2017). Isoniazid: A Review of Characteristics, Properties and Analytical Methods. Crit. Rev. Anal. Chem..

[54] Freire F.D., Aragão C.F.S., de Lima e Moura T.F.A., Raffin F.N. (2009). Thermal Studies of Isoniazid and Mixtures with Rifampicin. J. Therm. Anal. Calorim..

[55] Matos M.A.R., Miranda M.S., Morais V.M.F., Liebman J.F. (2005). Saccharin: A Combined Experimental and Computational Thermochemical Investigation of a Sweetener and Sulfonamide. Mol. Phys..

[56] Basavoju S., Boström D., Velaga S.P. (2008). Indomethacin-Saccharin Cocrystal: Design, Synthesis and Preliminary Pharmaceutical Characterization. Pharm. Res..

[57] Li D., Chen W., Zhou L., Xu L., He F., Heng J.Y.Y., Shehzad H., Ouyang J. (2024). Uncovering Cocrystal Formation and Competition Mechanism of Polyhydroxy Natural Products: Cases of Quercetin, Hesperidin, Resveratrol, and Curcumin. Cryst. Growth Des..

[58] (2023). APEX6: Software Suite for Crystallographic Data Collection and Reduction.

[59] Sheldrick G.M. (2015). SHELXT-Integrated Space-Group and Crystal-Structure Determination. Acta Cryst. A Found. Crystallogr..

[60] Sheldrick G.M. (2015). Crystal Structure Refinement with SHELXL. Acta Cryst. C Cryst. Struct. Commun..

[61] Dolomanov O.V., Bourhis L.J., Gildea R.J., Howard J.A.K., Puschmann H. (2009). OLEX2: A Complete Structure Solution, Refinement and Analysis Program. J. Appl. Crystallogr..

[62] Macrae C.F., Sovago I., Cottrell S.J., Galek P.T.A., McCabe P., Pidcock E., Platings M., Shields G.P., Stevens J.S., Towler M. (2020). Mercury 4.0: From Visualization to Analysis, Design and Prediction. J. Appl. Crystallogr..

[63] Döbelin N., Kleeberg R. (2015). Profex: A Graphical User Interface for the Rietveld Refinement Program BGMN. J. Appl. Crystallogr..

[64] Frisch M.J., Trucks G.W., Schlegel H.B., Scuseria G.E., Robb M.A., Cheeseman J.R., Scalmani G., Barone V., Petersson G.A., Nakatsuji H. (2016). Gaussian 16.

[65] Lee C., Yang W., Parr R.G. (1988). Development of the Colle–Salvetti Correlation-Energy Formula into a Functional of the Electron Density. Phys. Rev. B.

[66] Becke A.D. (1988). Density-Functional Exchange-Energy Approximation with Correct Asymptotic Behavior. Phys. Rev. A.

[67] Becke A.D. (1993). Density-Functional Thermochemistry. III. The Role of Exact Exchange. J. Chem. Phys..

[68] McLean A.D., Chandler G.S. (1980). Contracted Gaussian Basis Sets for Molecular Calculations. I. Second-Row Atoms, Z = 11–18. J. Chem. Phys..

[69] Krishnan R., Binkley J.S., Seeger R., Pople J.A. (1980). Self-Consistent Molecular Orbital Methods. XX. A Basis Set for Correlated Wave Functions. J. Chem. Phys..

[70] Binkley J.S., Pople J.A., Hehre W.J. (1980). Self-Consistent Molecular Orbital Methods. XXI. Small Split-Valence Basis Sets for First-Row and Second-Row Atoms. J. Am. Chem. Soc..

[71] Dovesi R., Erba A., Orlando R., Zicovich-Wilson C.M., Civalleri B., Maschio L., Rérat M., Casassa S., Baima J., Salustro S. (2018). Quantum-Mechanical Condensed Matter Simulations with CRYSTAL17. WIREs Comput. Mol. Sci..

[72] Hehre W.J., Ditchfield R., Pople J.A. (1972). Self-Consistent Molecular Orbital Methods. XII. Further Extensions of Gaussian-Type Basis Sets for Use in Molecular Orbital Studies of Organic Molecules. J. Chem. Phys..

[73] Hariharan P.C., Pople J.A. (1973). The Influence of Polarization Functions on Molecular Orbital Hydrogenation Energies. Theor. Chim. Acta.

[74] Grimme S., Ehrlich S., Goerigk L. (2011). Effect of the Damping Function in Dispersion-Corrected Density Functional Theory. J. Comput. Chem..

[75] Spackman P.R., Turner M.J., McKinnon J.J., Wolff S.K., Grimwood D.J., Jayatilaka D., Spackman M.A.J. (2021). CrystalExplorer: A program for Hirshfeld surface analysis, visualization and quantitative analysis of molecular crystals. Appl. Cryst..

