# Supplementary material for: Isoniazid–Saccharin Salts: Synthesis, Structural Aspects, Thermal Properties and Spectroscopic Characterization
Source: Molecules. 2026 Jun 22;31(12):2187. doi: 10.3390/molecules31122187 (PMC13306008; doi:10.3390/molecules31122187)
Supplement: Supplementary file 1 [file molecules-31-02187-s001.zip › molecules-4371236-supplementary.pdf]

Supplementary Material for

Isoniazid–Saccharin Co-crystals: Synthesis, Structural Aspects, Thermal Properties and Spectroscopic Characterization

Rezvan Mohammadi,[1,2] Ayberk Yilmaz,[1,3] Nihal Sarier,[1,4] José António Paixão,[5] Gulce Ogruc Ildiz[1,2] and Rui Fausto[1,2,6,*]

[1] *Spectroscopy@IKU, IKU-SPECTRA Molecular Sciences and Spectroscopy Applied Research Center, Istanbul Kultur University, Atakoy Campus, Bakirkoy 34156, Istanbul, Türkiye*

[2] *Department of Physics, Faculty of Sciences and Letters, Istanbul Kultur University, Atakoy Campus, Bakirkoy 34156, Istanbul, Türkiye*

[3] *Department of Physics, Science Faculty, Istanbul University, Vezneciler 34118, Istanbul, Türkiye*

[4] *Department of Civil Engineering, Faculty of Engineering, Istanbul Kultur University, Atakoy Campus, Bakirkoy 34156, Istanbul, Türkiye*

[5] *CFisUC, Department of Physics, University of Coimbra, 3004–516 Coimbra, Portugal*

[6] *CQC–IMS, Department of Chemistry, University of Coimbra, 3004–535 Coimbra, Portugal*

* Corresponding author: rfausto@ciuc.pt

INDEX

X-Ray structural data section:

INH-SAC mono-hydrated (MH) salt crystal:

Table S1 - Final coordinates and equivalent isotropic displacement parameters of the non-hydrogen atoms **S3**
Table S2 - Hydrogen atom positions and isotropic displacement parameters **S3**
Table S3 - Anisotropic displacement parameters **S4**
Table S4 - Bond distances **S4**
Table S5 - Bond angles **S5**
Table S6 - Torsion angles **S6**
Table S7 - Contact distances **S7**
Table S8 - Symmetry codes to equivalent positions **S9**

INH-SAC anhydrous (A) salt crystal:

Table S9 - Final coordinates and equivalent isotropic displacement parameters of the non-hydrogen atoms **S10**
Table S10 - Hydrogen atom positions and isotropic displacement parameters **S10**
Table S11 - Anisotropic displacement parameters **S11**
Table S12 - Bond distances **S11**
Table S13 - Bond angles **S12**
Table S14 - Torsion angles **S13**
Table S15 - Contact distances **S14**
Table S16 - Symmetry codes to equivalent positions **S15**

Table S17 - Cartesian coordinates for INH **S16**
Table S18 - Cartesian coordinates for SAC **S16**
Table S19 - Cartesian coordinates for INH/SAC **S16**
Table S20 - Cartesian coordinates for $(INH{+}H)^+$ **S17**
Table S21 - Cartesian coordinates for $(SAC{-}H)^-$ **S17**
Table S22 - Cartesian coordinates for SAC-OH **S18**
Table S23 - Cartesian coordinates for INH/(SAC-OH) **S18**

Table S24 - Assignment of the IR and Raman spectra of the anhydrous INH-SAC salt (A) **S19**
Table S25 - Assignment of the IR and Raman spectra of the monohydrated INH-SAC salt (MH) **S22**
Table S26 - Cartesian coordinates for HCBS **S24**
Table S27- Assignment of the IR and Raman spectra of HCBS **S25**
Figure S1 – Change of energy along geometry optimization for the $(INH+H)^+/(SAC–H)^–$ isolated dimer **S26**
Figure S2 – relative energies of the different INH and SAC isolated systems investigated in this study **S26**
Figure S3 - Hirshfeld surface for the $(INH+H)^+$ ion in the $(INH+H)^+/(SAC–H)^–$ salt (A) and 2D fingerprint plots for the different contacts **S27**
Figure S4 - Hirshfeld surface for the $(SAC–H)^–$ ion in the $(INH+H)^+/(SAC–H)^–$ salt (A) and 2D fingerprint plots for the different contacts **S28**
Figure S5 - Hirshfeld surface for the $(INH+H)^+$ ion in the $(INH+H)^+/(SAC–H)^–.H_2O$ salt (MH) and 2D fingerprint plots for the different contacts **S29**
Figure S6 - Hirshfeld surface for the $(SAC–H)^–$ ion in the $(INH+H)^+/(SAC–H)^–.H_2O$ salt (MH) and 2D fingerprint plots for the different contacts **S30**
Figure S7 - Hirshfeld surface for the water molecyle in the $(INH+H)^+/(SAC–H)^–.H_2O$ salt (MH) and 2D fingerprint plots for the different contacts **S29**
Figure S8 - DSC curves of pure INH (polymorph 1) and SAC **S31**
Figure S9 - Comparison of the experimental IR spectra of A and MH with the DFT calculated (periodic) spectra **S32**
Figure S10 - Comparison of the experimental Raman spectra of A and MH with the DFT calculated (periodic) spectra **S33**

Table S1: Final Coordinates (Å) and Equivalent Isotropic Displacement Parameters of the non-Hydrogen atoms for MH.

Atom	*x*	*y*	*z*	U(eq) [Å^2]
S1	0.44481(7)	0.49568(5)	0.18919(4)	0.0464(2)
O1	0.3314(2)	0.30157(18)	0.46886(10)	0.0586(5)
O2	0.2975(2)	0.55563(19)	0.12450(12)	0.0666(6)
O3	0.6108(2)	0.58211(17)	0.15686(13)	0.0662(6)
N1	0.3781(2)	0.49621(19)	0.31638(13)	0.0506(5)
C1	0.4921(2)	0.2768(2)	0.19467(14)	0.0374(5)
C2	0.5617(3)	0.1816(2)	0.11462(15)	0.0482(6)
C3	0.5875(3)	0.0101(2)	0.14637(17)	0.0534(7)
C4	0.5476(3)	-0.0624(2)	0.25330(17)	0.0501(6)
O4	-0.0025(2)	1.11196(18)	0.67513(12)	0.0637(5)
C5	0.4786(3)	0.0355(2)	0.33203(15)	0.0433(6)
C6	0.4501(2)	0.2064(2)	0.30141(13)	0.0358(5)
C7	0.3808(2)	0.3393(2)	0.37011(14)	0.0418(5)
N2	0.1823(2)	0.5522(2)	0.57034(13)	0.0488(5)
N3	-0.0654(2)	0.9460(2)	0.83342(14)	0.0525(6)
N4	-0.1324(3)	1.0809(3)	0.89018(19)	0.0725(8)
O5	0.9206(3)	0.6717(3)	0.00831(15)	0.0825(7)
C8	0.0987(3)	0.5221(3)	0.67095(17)	0.0540(7)
C9	0.0362(3)	0.6541(2)	0.72650(15)	0.0489(6)
C10	0.0594(2)	0.8171(2)	0.67669(14)	0.0387(5)
C11	0.1448(3)	0.8435(3)	0.57163(15)	0.0478(6)
C12	0.2051(3)	0.7081(3)	0.51973(16)	0.0517(7)
C13	-0.0053(2)	0.9715(2)	0.72902(15)	0.0425(6)

U(eq) = 1/3 of the trace of the orthogonalized U Tensor.

Table S2: Hydrogen Atom Positions (Å) and Isotropic Displacement Parameters for MH.

Atom	*x*	*y*	*z*	U(iso) [Å^2]
H2A	0.58982	0.23068	0.04301	0.0580
H3A	0.63274	-0.05859	0.09472	0.0640
H4	0.56745	-0.17836	0.27235	0.0600
H5	0.45208	-0.01302	0.40390	0.0520
H2	0.234(3)	0.458(3)	0.5354(18)	0.0590
H3	-0.062(3)	0.848(3)	0.873(2)	0.0630
H4A	-0.164(4)	1.157(4)	0.839(3)	0.0870
H4B	-0.035(4)	1.122(4)	0.903(3)	0.0870
H8	0.08258	0.41179	0.70378	0.0650
H9	-0.02124	0.63333	0.79701	0.0590
H11	0.16116	0.95258	0.53640	0.0570
H12	0.26215	0.72533	0.44899	0.0620
H5A	0.816(5)	0.614(5)	0.010(3)	0.1240
*H5B	1.021(5)	0.571(6)	0.020(5)	0.1240
*H5C	0.894(10)	0.721(10)	0.082(6)	0.1240

The temperature factor has the form of Exp(-*T*), Where $T = 8(\pi^2)U(\mathrm{Sin}(\theta)/\lambda)^2$ for isotropic atoms.

Table S3: (An)isotropic Displacement Parameters for MH.

Atom	U(1,1) or U	U(2,2)	U(3,3)	U(2,3)	U(1,3)	U(1,2)
S1	0.0656(3)	0.0290(3)	0.0416(3)	-0.0053(2)	0.0039(2)	0.0005(2)
O1	0.0868(10)	0.0510(8)	0.0345(7)	-0.0117(6)	0.0089(7)	0.0021(7)
O2	0.0865(11)	0.0516(9)	0.0550(9)	0.0035(7)	-0.0103(8)	0.0157(8)
O3	0.0856(11)	0.0406(8)	0.0696(10)	-0.0113(7)	0.0178(8)	-0.0192(7)
N1	0.0700(11)	0.0359(8)	0.0443(9)	-0.0135(6)	0.0044(8)	0.0033(7)
C1	0.0458(9)	0.0303(8)	0.0356(9)	-0.0067(7)	-0.0005(7)	-0.0019(7)
C2	0.0669(12)	0.0413(10)	0.0346(9)	-0.0101(7)	0.0052(8)	-0.0010(8)
C3	0.0728(13)	0.0395(10)	0.0491(11)	-0.0204(8)	0.0029(10)	0.0018(9)
C4	0.0663(12)	0.0290(9)	0.0551(12)	-0.0095(8)	-0.0048(9)	-0.0014(8)
O4	0.0894(11)	0.0432(8)	0.0543(9)	-0.0065(6)	0.0009(8)	0.0060(7)
C5	0.0556(11)	0.0352(9)	0.0382(9)	-0.0024(7)	-0.0032(8)	-0.0062(8)
C6	0.0406(9)	0.0338(8)	0.0335(8)	-0.0079(7)	-0.0017(7)	-0.0038(7)
C7	0.0493(10)	0.0400(9)	0.0362(9)	-0.0121(7)	0.0015(7)	0.0000(7)
N2	0.0535(9)	0.0508(9)	0.0442(9)	-0.0197(7)	-0.0029(7)	0.0038(7)
N3	0.0647(11)	0.0493(9)	0.0427(9)	-0.0169(7)	0.0038(8)	0.0060(8)
N4	0.0882(15)	0.0699(14)	0.0624(13)	-0.0367(10)	0.0009(11)	0.0129(12)
O5	0.0875(13)	0.0918(14)	0.0619(11)	-0.0026(10)	0.0025(9)	-0.0012(11)
C8	0.0683(13)	0.0446(11)	0.0476(11)	-0.0099(8)	0.0026(9)	-0.0021(9)
C9	0.0607(12)	0.0469(10)	0.0366(9)	-0.0088(8)	0.0055(8)	-0.0008(9)
C10	0.0362(8)	0.0458(10)	0.0349(9)	-0.0108(7)	-0.0049(7)	0.0025(7)
C11	0.0537(11)	0.0469(10)	0.0398(10)	-0.0062(8)	0.0028(8)	0.0017(8)
C12	0.0582(12)	0.0577(12)	0.0373(10)	-0.0113(9)	0.0037(8)	0.0010(9)
C13	0.0418(9)	0.0446(10)	0.0411(10)	-0.0107(8)	-0.0040(7)	0.0036(7)

The temperature factor has the form of Exp(-*T*), Where $T = 8(\pi^2)U(\mathrm{Sin}(\theta)/\lambda)^2$ for isotropic atoms and $T = 2(\pi^2)\Sigma_{ij}h(i)h(j)U(ij)A(i)^*A(j)^*$ for isotropic atoms. A(i)* are reciprocal axial lengths and h(i) are the reflection indices.

Table S4: Bond Distances (Å) for MH.

S1	-O2	1.4361(16)	N3	-N4	1.412(3)
S1	-O3	1.4404(16)	C4	-H4	0.9300
S1	-N1	1.6133(17)	C5	-H5	0.9300
S1	-C1	1.7587(17)	N2	-H2	0.96(2)
O1	-C7	1.246(2)	N3	-H3	0.86(2)
N1	-C7	1.335(2)	N4	-H4B	0.86(3)
C1	-C2	1.381(2)	N4	-H4A	0.85(3)
C1	-C6	1.380(2)	C8	-C9	1.377(3)
C2	-C3	1.379(2)	C9	-C10	1.378(2)
C3	-C4	1.385(3)	C10	-C13	1.510(2)
C4	-C5	1.382(3)	C10	-C11	1.383(3)
O4	-C13	1.224(2)	C11	-C12	1.371(3)
C5	-C6	1.374(2)	O5	-H5C	1.05(8)
C6	-C7	1.496(2)	O5	-H5A	0.93(4)
C2	-H2A	0.9300	O5	-H5B	1.05(5)
N2	-C8	1.332(3)	C8	-H8	0.9300
N2	-C12	1.330(3)	C9	-H9	0.9300
C3	-H3A	0.9300	C11	-H11	0.9300
N3	-C13	1.322(3)	C12	-H12	0.9300

Table S5: Bond Angles (Degrees) for MH.

O2	-S1	-O3	114.38(9)	C6	-C5	-H5	121.00
O2	-S1	-N1	110.92(9)	C4	-C5	-H5	121.00
O2	-S1	-C1	111.24(8)	C8	-N2	-H2	118.6(14)
O3	-S1	-N1	111.12(9)	C12	-N2	-H2	119.4(14)
O3	-S1	-C1	110.44(8)	N4	-N3	-H3	114.7(16)
N1	-S1	-C1	97.51(8)	C13	-N3	-H3	123.6(16)
S1	-N1	-C7	110.77(12)	H4A	-N4	-H4B	99(3)
S1	-C1	-C2	130.86(14)	N3	-N4	-H4B	104(2)
S1	-C1	-C6	106.55(12)	N3	-N4	-H4A	102(2)
C2	-C1	-C6	122.56(15)	N2	-C8	-C9	120.1(2)
C1	-C2	-C3	116.57(17)	C8	-C9	-C10	119.43(18)
C2	-C3	-C4	121.60(17)	C9	-C10	-C11	118.81(17)
C3	-C4	-C5	120.78(16)	C9	-C10	-C13	124.11(16)
C4	-C5	-C6	118.30(17)	C11	-C10	-C13	117.07(16)
C1	-C6	-C5	120.18(15)	C10	-C11	-C12	119.7(2)
C1	-C6	-C7	110.90(14)	N2	-C12	-C11	120.13(18)
C5	-C6	-C7	128.90(15)	O4	-C13	-N3	122.89(17)
O1	-C7	-N1	124.72(16)	O4	-C13	-C10	120.29(16)
O1	-C7	-C6	121.05(15)	N3	-C13	-C10	116.82(15)
N1	-C7	-C6	114.23(15)	H5A	-O5	-H5B	101(3)
C1	-C2	-H2A	122.00	H5A	-O5	-H5C	101(5)
C3	-C2	-H2A	122.00	N2	-C8	-H8	120.00
C8	-N2	-C12	121.86(18)	C9	-C8	-H8	120.00
C4	-C3	-H3A	119.00	C10	-C9	-H9	120.00
C2	-C3	-H3A	119.00	C8	-C9	-H9	120.00
N4	-N3	-C13	121.66(17)	C10	-C11	-H11	120.00
C3	-C4	-H4	120.00	C12	-C11	-H11	120.00
C5	-C4	-H4	120.00	C11	-C12	-H12	120.00
N2	-C12	-H12	120.00				

Table S6: Torsion Angles (Degrees) for MH.

O2	-S1	-N1	-C7	114.51(13)
O3	-S1	-N1	-C7	-117.07(12)
C1	-S1	-N1	-C7	-1.69(13)
O2	-S1	-C1	-C2	66.37(19)
O2	-S1	-C1	-C6	-115.25(12)
O3	-S1	-C1	-C2	-61.8(2)
O3	-S1	-C1	-C6	116.62(12)
N1	-S1	-C1	-C2	-177.68(18)
N1	-S1	-C1	-C6	0.70(12)
S1	-N1	-C7	-O1	-177.94(14)
S1	-N1	-C7	-C6	2.18(17)
S1	-C1	-C2	-C3	178.32(15)
C6	-C1	-C2	-C3	0.2(3)
S1	-C1	-C6	-C5	-177.95(14)
S1	-C1	-C6	-C7	0.39(15)
C2	-C1	-C6	-C5	0.6(3)
C2	-C1	-C6	-C7	178.94(16)
C1	-C2	-C3	-C4	-0.7(3)
C2	-C3	-C4	-C5	0.6(3)
C3	-C4	-C5	-C6	0.2(3)
C4	-C5	-C6	-C1	-0.8(3)
C4	-C5	-C6	-C7	-178.78(18)
C1	-C6	-C7	-O1	178.45(15)
C1	-C6	-C7	-N1	-1.68(19)
C5	-C6	-C7	-O1	-3.4(3)
C5	-C6	-C7	-N1	176.48(18)
C12	-N2	-C8	-C9	-1.3(3)
C8	-N2	-C12	-C11	1.2(3)
N4	-N3	-C13	-O4	0.5(3)
N4	-N3	-C13	-C10	-178.79(16)
N2	-C8	-C9	-C10	0.6(3)
C8	-C9	-C10	-C11	0.2(3)
C8	-C9	-C10	-C13	179.27(18)
C9	-C10	-C11	-C12	-0.4(3)
C13	-C10	-C11	-C12	-179.50(18)
C9	-C10	-C13	-O4	-170.04(18)
C9	-C10	-C13	-N3	9.2(2)
C11	-C10	-C13	-O4	9.1(2)
C11	-C10	-C13	-N3	-171.67(16)
C10	-C11	-C12	-N2	-0.3(3)

Table S7: Contact Distances (Å) for MH.

S1	.C6	2.5260(17)	N2	.O1	2.642(2)
S1	.H2A	3.0700	O2	.H5A_f	2.58(4)
O1	.N2	2.642(2)	N2	.C10	2.723(2)
O1	.C5	3.002(2)	O2	.H9_d	2.6400
O1	.C12_a	3.418(3)	O2	.H2A_e	2.6000
O2	.C7	3.392(2)	O2	.H4A_c	2.52(3)
O2	.C9_d	3.313(3)	O2	.H5B_b	2.53(4)
O2	.N4_c	3.056(3)	O3	.H5A	2.23(4)
O3	.O5	2.830(3)	O3	.H3A_g	2.8900
O3	.C4_g	3.255(2)	N3	.C9	2.886(2)
O3	.C7	3.418(2)	O3	.H4_g	2.5700
O3	.C3_g	3.418(2)	N3	.O5_m	2.859(3)
O3	.C9_a	3.416(3)	O3	.H5C	2.46(8)
O3	.C2	3.406(2)	O4	.H11	2.4600
O3	.C8_a	3.189(3)	O4	.H8_l	2.6300
O4	.C11_c	3.346(2)	N4	.O5_p	3.136(3)
O4	.C11	2.769(3)	N4	.O2_o	3.056(3)
O4	.C12_c	3.114(3)	O4	.H4B	2.86(4)
O4	.N4	2.733(3)	O4	.H4A	2.31(4)
O5	.O5_t	2.934(3)	O4	.H12_c	2.7300
O5	.N3_s	2.859(3)	N4	.O4	2.733(3)
O5	.O3	2.830(3)	O5	.H9_s	2.7000
O5	.N4_u	3.136(3)	O5	.H5A_t	2.89(4)
O1	.H2	1.68(2)	O5	.H3_s	2.04(2)
N1	.C6	2.379(2)	O5	.H4B_u	2.40(3)
O1	.H5	2.8200	C1	.C7	2.369(2)
N1	.C12	3.354(3)	N1	.H12	2.6900
N2	.C7	3.379(2)	C1	.C4	2.723(2)
N1	.H2	2.80(2)	C9	.C7_r	3.422(3)
C2	.O3	3.406(2)	C9	.N3	2.886(2)
C2	.C5	2.815(3)	C10	.N2	2.723(2)
N3	.H9	2.6100	C11	.C5_q	3.423(3)
C3	.O3_h	3.418(2)	C11	.C8	2.721(3)
C3	.C6	2.742(2)	C11	.C11_c	3.561(3)
C4	.O3_h	3.255(2)	C11	.O4	2.769(3)
C4	.C1	2.723(2)	C11	.C6_q	3.507(3)
N4	.H2A_n	2.9400	C11	.O4_c	3.346(2)
C4	.C13_a	3.542(3)	C12	.N1	3.354(3)
N4	.H5C_p	2.56(8)	C12	.O4_c	3.114(3)
C5	.O1	3.002(2)	C12	.C9	2.725(3)
C5	.C11_a	3.423(3)	C12	.O1_q	3.418(3)
C5	.C2	2.815(3)	C12	.C7_q	3.458(3)
C6	.C3	2.742(2)	C13	.C4_q	3.542(3)
C6	.N1	2.379(2)	C7	.H2	2.51(2)
C6	.C11_a	3.507(3)	C7	.H5	2.8100
C7	.O3	3.418(2)	C9	.H3	2.59(2)
C7	.N2	3.379(2)	C10	.H3	2.56(2)
C7	.O2	3.392(2)	C11	.H11_c	3.0000
C7	.C12_a	3.458(3)	C11	.H5_g	3.0700
C7	.C1	2.369(2)	C12	.H5_g	3.0800
C7	.C9_d	3.422(3)	C13	.H9	2.7300
C8	.O3_q	3.189(3)	C13	.H11	2.6000
C8	.C11	2.721(3)	C13	.H4A	2.34(3)

C9	.O2_r	3.313(3)	C13	.H4B	2.64(4)
C9	.C12	2.725(3)	H2	.N1	2.80(2)
C9	.O3_q	3.416(3)	H2	.O1	1.68(2)
H2	.H8	2.2600	H4B	.H5C_p	1.75(9)
H2	.H12	2.2700	H4B	.O4	2.86(4)
H2	.C7	2.51(2)	H5	.C7	2.8100
H2A	.N4_i	2.9400	H5	.O1	2.8200
H2A	.S1	3.0700	H5	.H11_k	2.5500
H2A	.H3A	2.3200	H5	.H12_k	2.5800
H2A	.O2_e	2.6000	H5	.C12_k	3.0800
H3	.C9	2.59(2)	H5	.H4	2.3200
H3	.C10	2.56(2)	H5	.C11_k	3.0700
H3	.O5_m	2.04(2)	H5A	.O5_t	2.89(4)
H3	.H5A_m	2.49(5)	H5A	.O3	2.23(4)
H3	.H9	2.0800	H5A	.H3_s	2.49(5)
H3	.H4A	2.52(4)	H5A	.O2_e	2.58(4)
H3	.H4B	2.33(4)	H5B	.H5A_t	1.88(6)
H3A	.O3_h	2.8900	H5B	.O5_t	2.04(5)
H3A	.H5C_j	2.5100	H5B	.O2_v	2.53(4)
H3A	.H4	2.3000	H5C	.O3	2.46(8)
H3A	.H2A	2.3200	H5C	.H3A_g	2.5100
H4	.H3A	2.3000	H5C	.N4_u	2.56(8)
H4	.O3_h	2.5700	H5C	.H4B_u	1.75(8)
H4	.H5	2.3200	H8	.O4_k	2.6300
H4A	.O4	2.31(4)	H8	.H2	2.2600
H4A	.C13	2.34(3)	H8	.H9	2.3100
H4A	.O2_o	2.52(3)	H9	.H8	2.3100
H4A	.H3	2.52(4)	H9	.O2_r	2.6400
H4B	.H3	2.33(4)	H9	.O5_m	2.7000
H4B	.C13	2.64(4)	H9	.N3	2.6100
H4B	.O5_p	2.40(3)	H9	.C13	2.7300
H9	.H3	2.0800	H12	.O4_c	2.7300
H11	.C13	2.6000	H12	.H5_g	2.5800
H11	.H5_g	2.5500	H12	.H11	2.3000
H11	.O4	2.4600	H12	.N1	2.6900
H11	.H12	2.3000	H12	.H2	2.2700
H11	.C11_c	3.0000			

Table S8: Translation of Symmetry Code to Equivalent Positions for MH.

a =[2666.00] = [2_666] = 1-x,1-y,1-z
b =[1455.00] = [1_455] = -1+x,y,z
c =[2576.00] = [2_576] = -x,2-y,1-z
d =[2566.00] = [2_566] = -x,1-y,1-z
e =[2665.00] = [2_665] = 1-x,1-y,-z
g =[1565.00] = [1_565] = x,1+y,z
h =[1545.00] = [1_545] = x,-1+y,z
i =[1644.00] = [1_644] = 1+x,-1+y,-1+z
l =[1565.00] = [1_565] = x,1+y,z
m =[1456.00] = [1_456] = -1+x,y,1+z
n =[1466.00] = [1_466] = -1+x,1+y,1+z
o =[2576.00] = [2_576] = -x,2-y,1-z
p =[2676.00] = [2_676] = 1-x,2-y,1-z
q =[2666.00] = [2_666] = 1-x,1-y,1-z
r =[2566.00] = [2_566] = -x,1-y,1-z
s =[1654.00] = [1_654] = 1+x,y,-1+z
t =[2765.00] = [2_765] = 2-x,1-y,-z
v =[1655.00] = [1_655] = 1+x,y,z

Table S9: Final Coordinates (Å) and Equivalent Isotropic Displacement Parameters of the non-Hydrogen atoms for A.

Atom	*x*	*y*	*z*	U(eq) [Å^2]
S1	0.82083(5)	0.44706(7)	0.32237(4)	0.0404(2)
O1	0.52371(15)	0.3265(2)	0.36314(12)	0.0510(6)
O2	0.83733(16)	0.5289(2)	0.24135(12)	0.0506(6)
O3	0.91051(17)	0.4827(2)	0.39670(13)	0.0600(7)
N1	0.68953(18)	0.4817(2)	0.34656(14)	0.0434(7)
C1	0.8056(2)	0.2266(3)	0.30583(15)	0.0369(7)
C2	0.8838(2)	0.1083(3)	0.28334(18)	0.0482(9)
C3	0.8424(2)	-0.0560(3)	0.27140(19)	0.0519(10)
C4	0.7261(2)	-0.0961(3)	0.27970(18)	0.0497(9)
O4	0.29474(15)	1.1825(2)	0.46294(14)	0.0577(7)
C5	0.6494(2)	0.0248(3)	0.30287(16)	0.0416(8)
C6	0.69116(19)	0.1876(3)	0.31721(14)	0.0341(7)
C7	0.6266(2)	0.3394(3)	0.34415(15)	0.0371(8)
N2	0.40991(18)	0.5950(3)	0.41206(13)	0.0421(7)
N3	0.12545(17)	1.0463(2)	0.47518(15)	0.0428(7)
N4	0.06367(19)	1.1899(3)	0.49856(18)	0.0471(8)
C8	0.3010(2)	0.5843(3)	0.43365(17)	0.0443(8)
C9	0.2396(2)	0.7289(3)	0.44892(16)	0.0416(8)
C10	0.29303(19)	0.8852(3)	0.44189(15)	0.0344(7)
C11	0.4062(2)	0.8905(3)	0.41938(16)	0.0424(8)
C12	0.4635(2)	0.7430(3)	0.40479(17)	0.0457(8)
C13	0.2373(2)	1.0525(3)	0.46036(15)	0.0362(7)

U(eq) = 1/3 of the trace of the orthogonalized U Tensor.

Table S10: Hydrogen Atom Positions (Å) and Isotropic Displacement Parameters for A.

Atom	*x*	*y*	*z*	U(iso) [Å^2]
H2A	0.96114	0.13694	0.27640	0.0580
H3A	0.89338	-0.14030	0.25763	0.0620
H4	0.69935	-0.20631	0.26949	0.0600
H5	0.57142	-0.00255	0.30870	0.0500
H2	0.451(2)	0.494(3)	0.3983(16)	0.0510
H3	0.083(2)	0.952(4)	0.4744(17)	0.056(8)
H4A	0.054(2)	1.264(4)	0.4519(18)	0.0570
H4B	0.110(2)	1.241(4)	0.5377(19)	0.0570
H8	0.26630	0.47909	0.43844	0.0530
H9	0.16339	0.72194	0.46373	0.0500
H11	0.44340	0.99369	0.41413	0.0510
H12	0.53973	0.74623	0.38982	0.0550

The temperature factor has the form of Exp(-*T*), Where $T = 8(\pi^2)U(\mathrm{Sin}(\theta)/\lambda)^2$ for isotropic atoms.

Table S11: (An)isotropic Displacement Parameters for A.

Atom	U(1,1) or U	U(2,2)	U(3,3)	U(2,3)	U(1,3)	U(1,2)
S1	0.0466(4)	0.0234(3)	0.0522(4)	-0.0029(2)	0.0103(3)	-0.0001(2)
O1	0.0458(10)	0.0381(10)	0.0740(13)	0.0019(8)	0.0249(9)	0.0095(8)
O2	0.0603(11)	0.0346(9)	0.0604(12)	0.0056(8)	0.0205(9)	-0.0050(8)
O3	0.0622(11)	0.0445(11)	0.0678(13)	-0.0114(9)	-0.0081(10)	-0.0046(9)
N1	0.0529(12)	0.0269(10)	0.0540(13)	-0.0007(9)	0.0197(10)	0.0060(9)
C1	0.0416(13)	0.0235(11)	0.0468(14)	-0.0001(9)	0.0107(11)	0.0023(9)
C2	0.0409(13)	0.0357(13)	0.0713(18)	-0.0009(12)	0.0196(13)	0.0049(11)
C3	0.0556(16)	0.0305(13)	0.073(2)	-0.0040(12)	0.0208(14)	0.0139(11)
C4	0.0611(16)	0.0231(12)	0.0672(18)	-0.0005(11)	0.0168(14)	0.0008(11)
O4	0.0493(10)	0.0286(9)	0.0993(15)	-0.0088(9)	0.0242(10)	-0.0048(8)
C5	0.0431(13)	0.0300(12)	0.0541(16)	0.0019(10)	0.0150(12)	-0.0004(10)
C6	0.0396(12)	0.0276(12)	0.0357(13)	0.0034(9)	0.0079(11)	0.0055(9)
C7	0.0440(13)	0.0289(12)	0.0402(14)	0.0028(9)	0.0121(11)	0.0088(10)
N2	0.0498(12)	0.0326(11)	0.0452(13)	-0.0012(9)	0.0115(10)	0.0131(10)
N3	0.0376(11)	0.0248(11)	0.0689(15)	-0.0074(9)	0.0171(10)	0.0025(9)
N4	0.0440(12)	0.0285(11)	0.0707(17)	-0.0078(10)	0.0144(12)	0.0045(9)
C8	0.0518(15)	0.0309(13)	0.0526(16)	0.0023(10)	0.0161(13)	0.0021(11)
C9	0.0433(13)	0.0305(12)	0.0538(16)	0.0009(10)	0.0169(12)	0.0031(10)
C10	0.0387(12)	0.0291(12)	0.0357(13)	0.0001(9)	0.0068(10)	0.0028(10)
C11	0.0427(13)	0.0326(13)	0.0537(16)	-0.0036(11)	0.0134(12)	-0.0013(11)
C12	0.0410(13)	0.0424(15)	0.0558(16)	-0.0025(11)	0.0142(12)	0.0055(11)
C13	0.0389(12)	0.0285(12)	0.0413(14)	-0.0026(9)	0.0068(11)	0.0021(10)

The temperature factor has the form of Exp(-T), Where $T = 8(\pi^2)U(Sin(\theta)/\lambda)^2$ for isotropic atoms and $T = 2(\pi^2)\Sigma_{ij}h(i)h(j)U(ij)A(i)^*A(j)^*$ for isotropic atoms. A(i)* are reciprocal axial lengths and h(i) are the reflection indices.

Table S512: Bond Distances (Å) for A.

S1	-O2	1.4387(19)	N3	-C13	1.327(3)
S1	-O3	1.439(2)	N3	-N4	1.412(3)
S1	-N1	1.616(2)	C4	-H4	0.9300
S1	-C1	1.770(2)	C5	-H5	0.9300
O1	-C7	1.252(3)	N2	-H2	0.97(2)
N1	-C7	1.333(3)	N3	-H3	0.89(3)
C1	-C2	1.372(3)	N4	-H4B	0.84(3)
C1	-C6	1.374(3)	N4	-H4A	0.92(3)
C2	-C3	1.387(3)	C8	-C9	1.380(3)
C3	-C4	1.385(3)	C9	-C10	1.391(3)
C4	-C5	1.378(3)	C10	-C13	1.515(3)
O4	-C13	1.217(3)	C10	-C11	1.383(3)
C5	-C6	1.381(3)	C11	-C12	1.373(3)
C6	-C7	1.499(3)	C8	-H8	0.9300
C2	-H2A	0.9300	C9	-H9	0.9300
N2	-C8	1.332(3)	C11	-H11	0.9300
N2	-C12	1.334(3)	C12	-H12	0.9300
C3	-H3A	0.9300			

Table S13: Bond Angles (Degrees) for A.

O2	-S1	-O3	115.27(11)	C6	-C5	-H5	121.00
O2	-S1	-N1	111.51(11)	C4	-C5	-H5	121.00
O2	-S1	-C1	110.11(10)	C8	-N2	-H2	120.0(14)
O3	-S1	-N1	110.77(11)	C12	-N2	-H2	117.9(14)
O3	-S1	-C1	110.47(10)	N4	-N3	-H3	113.0(17)
N1	-S1	-C1	97.28(10)	C13	-N3	-H3	124.2(17)
S1	-N1	-C7	111.16(15)	H4A	-N4	-H4B	104(3)
S1	-C1	-C2	131.10(19)	N3	-N4	-H4B	106.7(19)
S1	-C1	-C6	106.14(17)	N3	-N4	-H4A	108.9(17)
C2	-C1	-C6	122.7(2)	N2	-C8	-C9	120.2(2)
C1	-C2	-C3	117.0(2)	C8	-C9	-C10	119.2(2)
C2	-C3	-C4	120.9(2)	C9	-C10	-C11	118.7(2)
C3	-C4	-C5	121.0(2)	C9	-C10	-C13	124.4(2)
C4	-C5	-C6	118.3(2)	C11	-C10	-C13	116.9(2)
C1	-C6	-C5	120.0(2)	C10	-C11	-C12	119.8(2)
C1	-C6	-C7	111.5(2)	N2	-C12	-C11	120.1(2)
C5	-C6	-C7	128.5(2)	O4	-C13	-N3	123.4(2)
O1	-C7	-N1	125.4(2)	O4	-C13	-C10	120.6(2)
O1	-C7	-C6	120.8(2)	N3	-C13	-C10	116.0(2)
N1	-C7	-C6	113.8(2)	N2	-C8	-H8	120.00
C1	-C2	-H2A	121.00	C9	-C8	-H8	120.00
C3	-C2	-H2A	121.00	C8	-C9	-H9	120.00
C8	-N2	-C12	122.0(2)	C10	-C9	-H9	120.00
C4	-C3	-H3A	120.00	C10	-C11	-H11	120.00
C2	-C3	-H3A	120.00	C12	-C11	-H11	120.00
N4	-N3	-C13	122.72(18)	N2	-C12	-H12	120.00
C3	-C4	-H4	120.00	C11	-C12	-H12	120.00
C5	-C4	-H4	119.00				

Table S14: Torsion Angles (Degrees) for A.

O2	-S1	-N1	-C7	114.81(17)
O3	-S1	-N1	-C7	-115.38(17)
C1	-S1	-N1	-C7	-0.20(19)
O2	-S1	-C1	-C2	64.1(3)
O2	-S1	-C1	-C6	-113.83(17)
O3	-S1	-C1	-C2	-64.3(3)
O3	-S1	-C1	-C6	117.72(17)
N1	-S1	-C1	-C2	-179.7(2)
N1	-S1	-C1	-C6	2.31(18)
S1	-N1	-C7	-O1	177.2(2)
S1	-N1	-C7	-C6	-1.9(3)
S1	-C1	-C2	-C3	-177.1(2)
C6	-C1	-C2	-C3	0.5(4)
S1	-C1	-C6	-C5	175.83(18)
S1	-C1	-C6	-C7	-3.5(2)
C2	-C1	-C6	-C5	-2.3(4)
C2	-C1	-C6	-C7	178.3(2)
C1	-C2	-C3	-C4	1.7(4)
C2	-C3	-C4	-C5	-2.2(4)
C3	-C4	-C5	-C6	0.3(4)
C4	-C5	-C6	-C1	1.8(3)
C4	-C5	-C6	-C7	-178.9(2)
C1	-C6	-C7	-O1	-175.4(2)
C1	-C6	-C7	-N1	3.7(3)
C5	-C6	-C7	-O1	5.4(4)
C5	-C6	-C7	-N1	-175.6(2)
C12	-N2	-C8	-C9	0.3(4)
C8	-N2	-C12	-C11	-0.3(4)
N4	-N3	-C13	-O4	-2.3(4)
N4	-N3	-C13	-C10	176.1(2)
N2	-C8	-C9	-C10	-0.3(4)
C8	-C9	-C10	-C11	0.3(3)
C8	-C9	-C10	-C13	-177.5(2)
C9	-C10	-C11	-C12	-0.2(3)
C13	-C10	-C11	-C12	177.7(2)
C9	-C10	-C13	-O4	171.3(2)
C9	-C10	-C13	-N3	-7.2(3)
C11	-C10	-C13	-O4	-6.5(3)
C11	-C10	-C13	-N3	175.0(2)
C10	-C11	-C12	-N2	0.2(4)

Table S15: Contact Distances (Å) for A.

S1	.C6	2.525(2)	O2	.H2A_c	2.5000
S1	.H2A	3.0700	N2	.O1	2.657(3)
S1	.H4_a	3.1300	N3	.N4_m	2.925(3)
O1	.N2	2.657(3)	N3	.C9	2.886(3)
O1	.C5	3.002(3)	N3	.N3_m	3.149(3)
O1	.C5_b	3.365(3)	O3	.H9_g	2.9100
O1	.C4_b	3.374(3)	O3	.H4B_f	2.44(3)
O2	.C3_a	3.321(3)	O3	.H4A_e	2.44(3)
O2	.C7	3.409(3)	O4	.H8_l	2.3900
O2	.C10_d	3.185(3)	O4	.H12_f	2.7700
O2	.C4_a	3.317(3)	O4	.H11	2.4600
O2	.C2_c	3.311(3)	N4	.N3_m	2.925(3)
O2	.C13_d	3.088(3)	O4	.H4B	2.58(2)
O3	.N4_f	3.042(3)	N4	.O3_n	3.169(3)
O3	.C7	3.404(3)	N4	.O4	2.764(3)
O3	.N4_e	3.169(3)	N4	.O3_o	3.042(3)
O4	.C8_l	3.218(3)	O4	.H4A	2.79(2)
O4	.C11	2.768(3)	C1	.C7	2.377(3)
O4	.C12_f	3.217(3)	N1	.H4_a	2.7500
O4	.N4	2.764(3)	N1	.H2	2.94(2)
O1	.H2	1.69(2)	C1	.C4	2.722(3)
O1	.H5	2.8200	N1	.H12	2.8400
N1	.C8_g	3.397(3)	C2	.C5	2.807(3)
N1	.C6	2.374(3)	C2	.O2_h	3.311(3)
N2	.C5_b	3.311(3)	C3	.C8_d	3.499(4)
N2	.C10	2.729(3)	N3	.H3_m	2.60(2)
O2	.H3A_a	2.7000	C3	.O2_i	3.321(3)
O2	.H4_a	2.6900	N3	.H9	2.6200
C3	.C6	2.746(3)	C10	.O2_b	3.185(3)
C4	.O2_i	3.317(3)	C10	.N2	2.729(3)
N4	.H3_m	2.10(3)	C11	.C11_f	3.478(3)
C4	.C1	2.722(3)	C11	.C8	2.729(3)
C4	.O1_j	3.374(3)	C11	.O4	2.768(3)
C4	.C8_d	3.542(4)	C12	.O4_f	3.217(3)
N4	.H9_m	2.8200	C12	.C5_a	3.591(3)
C5	.O1	3.002(3)	C12	.C9	2.734(3)
C5	.C12_k	3.591(3)	C13	.O2_b	3.088(3)
C5	.O1_j	3.365(3)	C5	.H12_k	2.9500
C5	.C2	2.807(3)	C7	.H5	2.8200
C5	.N2_d	3.311(3)	C7	.H2	2.58(2)
C6	.N1	2.374(3)	C9	.H3	2.58(3)
C6	.C3	2.746(3)	C10	.H3	2.57(2)
C7	.C1	2.377(3)	C11	.H5_a	2.8400
C7	.O2	3.409(3)	C11	.H11_f	3.0000
C7	.O3	3.404(3)	C12	.H5_a	2.8800
C7	.C9_g	3.351(3)	C13	.H4B	2.50(3)
C7	.C8_g	3.437(3)	C13	.H9	2.7500
C8	.C7_p	3.437(3)	C13	.H4A	2.66(3)
C8	.C11	2.729(3)	C13	.H11	2.5900
C8	.O4_k	3.218(3)	H2	.H12	2.2500
C8	.N1_p	3.397(3)	H2	.C7	2.58(2)
C8	.C4_b	3.542(4)	H2	.H8	2.2800
C8	.C3_b	3.499(4)	H2	.O1	1.69(2)

C9	.N3	2.886(3)	H2	.N1	2.94(2)
C9	.C12	2.734(3)	H2A	.S1	3.0700
C9	.C7_p	3.351(3)	H2A	.H3A	2.3300
H2A	.O2_h	2.5000	H5	.C7	2.8200
H2A	.H3A_c	2.5300	H5	.C11_k	2.8400
H3	.C9	2.58(3)	H5	.C12_k	2.8800
H3	.H4A	2.51(4)	H5	.H4	2.3100
H3	.H4B	2.49(4)	H5	.H11_k	2.3300
H3	.H9	2.0600	H5	.H12_k	2.4000
H3	.N3_m	2.60(2)	H5	.O1	2.8200
H3	.N4_m	2.10(3)	H8	.O4_k	2.3900
H3	.H3_m	2.28(3)	H8	.H2	2.2800
H3	.C10	2.57(2)	H8	.H9	2.3200
H3A	.O2_i	2.7000	H9	.N3	2.6200
H3A	.H2A	2.3300	H9	.C13	2.7500
H3A	.H4	2.3000	H9	.H3	2.0600
H3A	.H2A_h	2.5300	H9	.H8	2.3200
H4	.S1_i	3.1300	H9	.O3_p	2.9100
H4	.O2_i	2.6900	H9	.N4_m	2.8200
H4	.N1_i	2.7500	H11	.O4	2.4600
H4	.H3A	2.3000	H11	.C13	2.5900
H4	.H5	2.3100	H11	.H5_a	2.3300
H4A	.O3_n	2.44(3)	H11	.H12	2.3000
H4A	.O4	2.79(2)	H11	.C11_f	3.0000
H4A	.C13	2.66(3)	H12	.N1	2.8400
H4A	.H3	2.51(4)	H12	.C5_a	2.9500
H4B	.O4	2.58(2)	H12	.H2	2.2500
H4B	.C13	2.50(3)	H12	.H5_a	2.4000
H4B	.H3	2.49(4)	H12	.H11	2.3000
H4B	.O3_o	2.44(3)	H12	.O4_f	2.7700

Table S16: Translation of Symmetry Code to Equivalent Positions for A.

a =[1565.00] = [1_565] = x,1+y,z
b =[2655.00] = [2_655] = 1-x,1/2+y,1/2-z
c =[2755.00] = [2_755] = 2-x,1/2+y,1/2-z
d =[2645.00] = [2_645] = 1-x,-1/2+y,1/2-z
e =[1645.00] = [1_645] = 1+x,-1+y,z
f =[3676.00] = [3_676] = 1-x,2-y,1-z
g =[3666.00] = [3_666] = 1-x,1-y,1-z
h =[2745.00] = [2_745] = 2-x,-1/2+y,1/2-z
i =[1545.00] = [1_545] = x,-1+y,z
j =[2645.00] = [2_645] = 1-x,-1/2+y,1/2-z
k =[1545.00] = [1_545] = x,-1+y,z
l =[1565.00] = [1_565] = x,1+y,z
m =[3576.00] = [3_576] = -x,2-y,1-z
n =[1465.00] = [1_465] = -1+x,1+y,z
o =[3676.00] = [3_676] = 1-x,2-y,1-z
p =[3666.00] = [3_666] = 1-x,1-y,1-z

Table S17 – Cartesian coordinates (Å) for INH.

	x	*y*	*z*
8	1.701735209	1.447203714	0.437318895
7	-3.019460993	-0.244016073	-0.075961924
7	3.443585810	-0.567319123	-0.279644916
7	2.045303009	-0.707661480	-0.186229849
6	-0.237495198	0.103329309	0.052717499
6	1.243339301	0.359991147	0.124059222
6	-2.459578578	0.961545897	-0.225424870
1	-3.143069173	1.786945039	-0.401404007
6	-2.200845067	-1.274566485	0.148400473
1	-2.674508860	-2.242645969	0.283915388
6	-1.087878726	1.188319287	-0.162803793
1	-0.675515735	2.183228952	-0.273248691
6	-0.813537475	-1.158278567	0.219448518
1	-0.213494417	-2.034741049	0.434135410
1	1.670770953	-1.475507030	-0.722168086
1	3.641107241	0.370569895	-0.624891602
1	3.830808003	-0.614546367	0.659584954

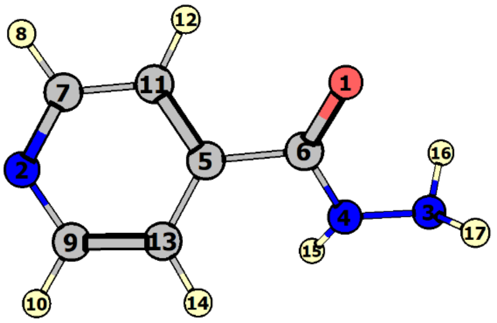

Table S18 – Cartesian coordinates (Å) for SAC.

	x	*y*	*z*
6	0.554277000	0.926443000	0.000000000
6	-0.638298000	0.210816000	0.000000000
6	-1.880429000	0.822863000	0.000000000
6	-1.900114000	2.219393000	0.000000000
6	-0.711900000	2.956208000	0.000000000
6	0.527130000	2.316351000	0.000000000
6	1.777178000	0.068306000	0.000000000
1	-2.797332000	0.246900000	0.000000000
1	-2.852312000	2.736610000	0.000000000
1	-0.756251000	4.038832000	0.000000000
1	1.456849000	2.872139000	0.000000000
1	2.035135000	-2.026278000	0.000000000
16	-0.318352000	-1.554032000	0.000000000
8	2.926372000	0.442773000	0.000000000
7	1.374272000	-1.260301000	0.000000000
8	-0.711900000	-2.177878000	1.255061000
8	-0.711900000	-2.177878000	-1.255061000

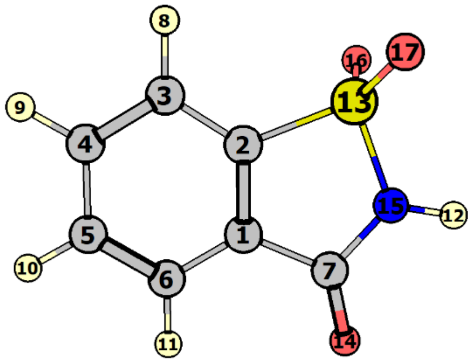

Table S19 – Cartesian coordinates (Å) for INH/SAC.

	x	*y*	*z*
6	3.530802000	0.989698000	-0.069676000
6	3.965872000	-0.329842000	-0.046430000
6	5.304273000	-0.679691000	-0.110090000
6	6.229575000	0.362545000	-0.201777000
6	5.810664000	1.696436000	-0.226897000
6	4.456200000	2.022855000	-0.161054000
6	2.043172000	1.128994000	0.012069000
1	5.621930000	-1.714733000	-0.089613000
1	7.287067000	0.131296000	-0.253850000
1	6.549958000	2.485571000	-0.298111000
1	4.114954000	3.050788000	-0.178557000
1	0.439544000	-0.266695000	0.141322000
8	1.421397000	2.172811000	-0.004616000
7	1.476961000	-0.124673000	0.110039000
16	2.558840000	-1.438378000	0.074900000
8	2.373494000	-2.209489000	-1.149833000
8	2.539869000	-2.143981000	1.351028000
6	-2.213128000	-1.243958000	0.208219000

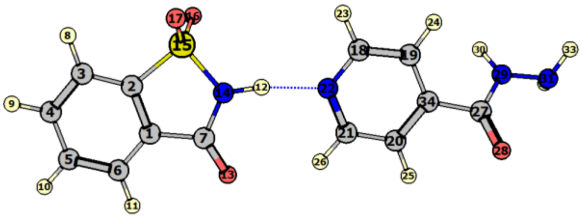

6	-3.596374000	-1.090647000	0.187837000
6	-3.245122000	1.277406000	0.102470000
6	-1.877766000	1.028025000	0.112917000
7	-1.366688000	-0.210856000	0.162502000
1	-1.767024000	-2.231810000	0.267423000
1	-4.229700000	-1.966886000	0.256218000
1	-3.628304000	2.289539000	0.083773000
1	-1.153363000	1.836635000	0.082886000
6	-5.613712000	0.496800000	0.130622000
8	-6.052305000	1.507583000	0.635397000
7	-6.377783000	-0.471485000	-0.480359000
1	-5.911054000	-1.159800000	-1.056596000
7	-7.764717000	-0.327048000	-0.567706000
1	-8.055558000	-0.093812000	-1.511021000
1	-8.227913000	-1.168942000	-0.247816000
6	-4.130461000	0.199037000	0.124856000

Table S20 – Cartesian coordinates (Å) for $(INH{+}H)^{+}$.

	x	*y*	*z*
6	-0.778780000	-1.179773000	0.213050000
6	-0.193752000	0.077401000	0.016906000
6	-1.018700000	1.189308000	-0.196718000
6	-2.385705000	1.024306000	-0.238290000
6	-2.154012000	-1.301512000	0.175373000
1	-0.184375000	-2.059402000	0.422798000
1	-0.578260000	2.171396000	-0.311218000
1	-3.085009000	1.832256000	-0.406965000
1	-3.921147000	-0.314499000	-0.078364000
1	-2.677656000	-2.236037000	0.326398000
7	-2.910951000	-0.208584000	-0.053630000
7	2.115687000	-0.681800000	-0.231999000
7	3.513425000	-0.546747000	-0.266235000
8	1.677185000	1.431937000	0.508679000
6	1.299481000	0.344630000	0.118299000
1	1.765474000	-1.463992000	-0.767071000
1	3.746899000	0.385060000	-0.602334000
1	3.878271000	-0.636528000	0.678652000

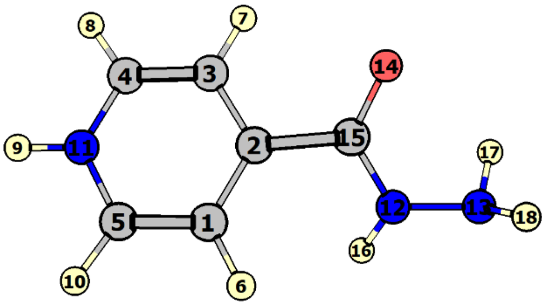

Table S21 – Cartesian coordinates (Å) for $(SAC{-}H)^{-}$.

	x	*y*	*z*
6	0.306210000	-0.607858000	0.000803000
6	0.787834000	0.689620000	-0.000041000
6	2.158852000	0.921710000	-0.000441000
6	3.022535000	-0.176459000	-0.000270000
6	2.519020000	-1.482504000	0.000355000
6	1.140231000	-1.714752000	0.000766000
6	-0.335262000	1.722941000	0.000218000
1	2.527157000	1.941561000	-0.000850000
1	4.096748000	-0.019512000	-0.000783000
1	3.205769000	-2.323178000	0.000475000
1	0.738868000	-2.721731000	0.001318000
7	-1.567021000	1.161254000	0.000544000
8	-0.076992000	2.929317000	0.000365000
16	-1.504269000	-0.474048000	-0.000154000
8	-2.030907000	-1.060376000	-1.248173000
8	-2.033052000	-1.061108000	1.246579000

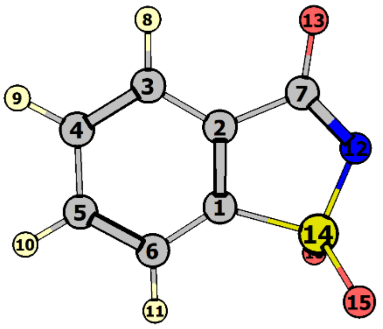

Table S22 – Cartesian coordinates (Å) for SAC-OH.

	x	*y*	*z*
6	0.693966000	0.202107000	0.000000000
6	-0.511999000	0.893889000	0.000000000
6	-0.527034000	2.284586000	0.000000000
6	0.699851000	2.951198000	0.000000000
6	1.903688000	2.241762000	0.000000000
6	1.917870000	0.842642000	0.000000000
6	-1.637617000	-0.063647000	0.000000000
1	-1.463194000	2.828700000	0.000000000
1	0.719612000	4.034504000	0.000000000
1	2.842792000	2.782730000	0.000000000
1	2.848187000	0.288563000	0.000000000
16	0.306084000	-1.567685000	0.000000000
7	-1.395672000	-1.323719000	0.000000000
8	0.699851000	-2.178330000	1.260741000
8	0.699851000	-2.178330000	-1.260741000
8	-2.875318000	0.432400000	0.000000000
1	-3.502449000	-0.306644000	0.000000000

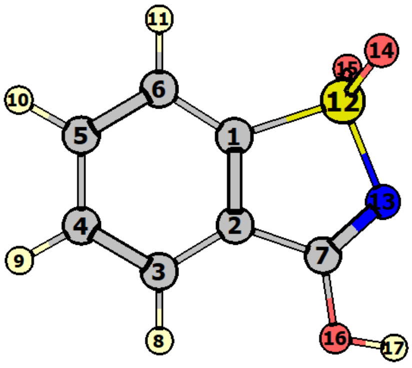

Table S23 – Cartesian coordinates (Å) for INH/(SAC-OH).

	x	*y*	*z*
16	4.023065000	4.125863000	2.206123000
8	3.212874000	3.095806000	5.713117000
8	5.422763000	4.381384000	1.886377000
8	2.982359000	4.817586000	1.454100000
7	3.768971000	4.359242000	3.874753000
6	3.695373000	2.346968000	2.257478000
6	3.465787000	3.230695000	4.434701000
7	3.371358000	5.403443000	7.042675000
1	3.281287000	3.992194000	6.204097000
6	3.532620000	7.844040000	8.380222000
6	3.839985000	7.750568000	7.022957000
1	4.156166000	8.630460000	6.477945000
6	3.738472000	6.516844000	6.390869000
1	3.952135000	6.391733000	5.334866000
6	3.095512000	5.486084000	8.349102000
1	2.818993000	4.560629000	8.843684000
6	3.708069000	1.408066000	1.243189000
6	3.408940000	0.087611000	1.594518000
6	3.401612000	2.021573000	3.575222000
6	3.104098000	0.709036000	3.924986000
1	2.875080000	0.451782000	4.951617000
6	3.111468000	-0.256337000	2.916165000
1	3.939464000	1.680789000	0.221051000
1	3.408340000	-0.679705000	0.828971000
1	2.883568000	-1.287371000	3.159971000
6	3.159062000	6.680223000	9.057979000
1	2.951030000	6.681938000	10.120840000
6	3.659042000	9.188490000	9.049596000
8	4.429423000	10.041427000	8.642924000
7	2.785727000	10.631550000	10.786597000
1	3.586463000	10.713854000	11.407996000
1	2.891378000	11.357716000	10.080096000
7	2.854805000	9.382170000	10.141290000
1	2.038908000	8.803764000	10.273191000

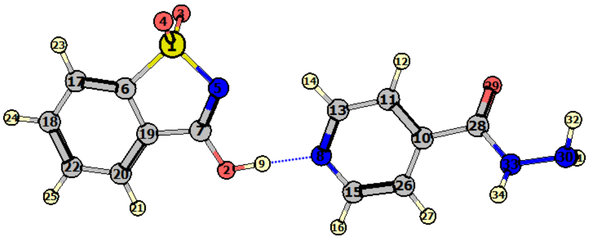

Table S24. Assignment of the IR and Raman spectra for INH-SAC anhydrous salt (A).[a]

A Exp.		A; Fully Periodic B3LYP/6-31G(d,p)-D3									
		A$_u$		*B*$_u$		*A*$_g$		*B*$_g$			
$\tilde{\nu}$ (IR)	$\tilde{\nu}$ (Raman)	$\tilde{\nu}$	I_{IR}	$\tilde{\nu}$	I_{IR}	$\tilde{\nu}$	a_R[b]	$\tilde{\nu}$	a_R[b]	DS[c]	Assignment[d]
3328		3423	5	3424	272	3423	66	3424	0	1	νNH_2 as
3305		3389	16	3389	180	3389	67	3389	1	0	νNH_2 s
3261, 3228	3227	3301	0	3301	3	3301	133	3301	59	1	νNH
3156		3168	13	3168	311	3167	182	3168	1	0	νCH (INH)
3088	3080	3126	530	3126	77	3125	216	3125	44	1	νCH (SAC)
	3074	3123	232	3123	25	3124	22	3124	16	1	νCH (INH)
3056	3059	3108	22	3107	4	3108	95	3107	24	1	νCH (SAC)
3031	3051	3092	1627	3093	4888	3077	103	3077	44	16	νCH (SAC)
3015	3016	2988	122	2988	0	2988	163	2988	10	0	νCH (INH)
2963	2983	2979	134	2978	429	2976	190	2976	114	3	νCH (SAC)
2764		2741	11726	2745	9466	2728	100	2737	100	18	νCH (INH)
2514	2569	2651	255	2651	263	2651	247	2651	27	0	νNH(···O=)
1680	1689	1776	216	1784	4764	1775	62	1782	5	9	νC=O (SAC)
	1681	1725	1	1725	36	1727	46	1725	45	3	δNH(···O=)
	1661	1696	11	1697	27	1696	10	1697	44	2	νCC (INH)
		1686	197	1686	1388	1695	45	1696	61	9	νC=O (INH)
1646	1651	1683	558	1683	512	1683	86	1684	46	2	δNH_2
1639	1636	1670	286	1670	1907	1675	17	1674	54	5	νCC (SAC)
1611	1601	1620	803	1620	219	1629	18	1628	8	9	δNH
1570	1588	1593	21	1595	153	1594	7	1596	16	2	νCC (SAC)
1561	1579	1591	0	1591	52	1589	20	1592	0	2	νCC (INH)
1543	1554	1560	124	1560	404	1550	1	1558	4	10	δCH (INH)
1519	1501	1510	24	1512	44	1512	17	1512	2	2	νCC (SAC)
1496	1481	1477	2	1476	98	1477	6	1476	4	1	δCH (SAC)
1457,1446	1456	1423	82	1423	39	1427	298	1426	17	4	νCC (INH)
1415	1407	1391	836	1392	37	1408	146	1409	7	17	νCC (SAC)
1388	1367	1386	443	1388	80	1388	67	1386	4	2	δCH (INH)
1359		1371	11	1370	27	1369	20	1370	11	2	δCH (INH)
1350		1368	201	1368	92	1364	52	1363	64	5	$twNH_2$
1339	1336	1326	453	1325	136	1326	97	1326	17	1	νCN (SAC)
1326	1326	1315	109	1315	63	1314	47	1315	20	1	νCN (INH)
1285	1292	1277	696	1276	1063	1276	63	1282	5	6	νSO_2 as
1254	1258	1242	1063	1253	388	1242	216	1254	27	12	δCH (INH)
1247	1223	1238	14	1239	0	1240	5	1242	5	4	νCC (INH)
1214	1206	1210	61	1210	4	1212	28	1212	43	2	δCH (SAC)
		1204	32	1199	388	1205	8	1200	11	6	δCH (SAC)
1148	1179	1169	33	1169	735	1158	44	1161	5	10	δCH (SAC)
1135	1159	1155	0	1160	319	1154	21	1157	3	6	γNH(···O=)

1112	1117	1119	0	1117	316	1130	6	1132	9	15	νCC (SAC)
		1111	4	1111	39	1114	47	1114	3	3	νNN
1101	1098	1094	28	1097	161	1099	35	1099	2	5	νCC (INH)
		1092	17	1093	255	1093	13	1095	0	3	νSO_2 s
	1070	1089	58	1089	42	1090	15	1091	17	2	νCN (INH)
1055	1062	1058	16	1057	90	1057	112	1057	2	1	νCS
1022	1038	1025	10	1026	87	1025	113	1024	3	2	νCC (SAC)
	1013	1025	53	1018	19	1025	65	1019	1	6	γCH (SAC)
984	1000	1011	41	1010	1	1011	6	1011	1	1	δring (INH
955	965	969	89	970	802	976	1	977	2	8	γCH (INH)
		966	5	966	149	969	6	970	6	5	νNS
		951	0	952	307	952	4	952	6	1	γCH (INH)
928	933	929	14	927	1148	928	1	914	3	15	γCH (SAC)
		927	97	912	174	906	44	906	21	21	wNH_2
877	872	892	54	893	113	890	7	894	1	3	γCH (SAC)
858	851	882	40	880	231	837	40	838	22	45	γCH (INH)
818		784	14	783	66	792	54	793	24	10	δring (INH)
798	787	780	252	780	266	780	70	781	8	1	γCH (INH)
756	764	767	45	764	475	768	31	768	8	4	δring (SAC)
735	736	749	416	751	967	754	54	761	7	12	γCH (SAC)
	720	730	0	731	25	730	6	732	20	1	γNH
703	704	704	53	710	153	707	124	713	16	10	τring (INH)
678	689	697	29	697	292	702	43	705	62	9	τring (SAC)
		677	1	677	8	686	5	685	1	9	δring (SAC)
	664	674	8	673	156	675	211	674	6	1	δring (INH)
		669	0	659	794	663	38	664	9	10	γC=O (INH)
654	654	651	0	652	2	650	54	651	12	1	γC=O (SAC)
635	632	643	69	641	104	641	9	641	1	3	δring (INH)
		640	7	636	655	637	3	639	9	3	wC=O (SAC)
598	604	611	4	610	3	611	19	610	3	2	δSO_2
	589	583	13	584	293	583	70	586	2	3	γSO_2
538	539	534	141	536	62	534	39	536	23	3	τring (SAC)
525	529	520	189	523	171	520	11	523	25	3	wSO_2
478	491	492	4	494	86	495	29	497	3	5	τring (INH)
453	451	488	128	488	19	491	84	492	6	5	τring (INH)
418	412	422	24	422	81	422	22	423	9	1	τring (SAC)
		407	1	404	26	407	11	406	1	3	τring (INH)
400[e]	392	400	25	402	69	403	3	403	3	3	τring (INH)
	382	395	78	395	53	393	53	393	25	3	δring (SAC)
		379	161	381	314	373	88	377	35	8	δring (SAC)
	358	366	150	371	10	365	91	366	3	6	δOCN, δCNN (INH)
	344	354	212	354	12	352	33	352	16	3	δCNN, δOCN (INH)
	326	327	2	326	38	324	5	325	3	3	τring (SAC)
	298	280	3	278	12	281	60	280	7	2	τCN (SAC)
	274	255	2	254	4	255	79	254	23	1	twSO_2, τNN,

261	246	9	243	27	252	91	253	13	9	γC-C(ONHNH$_2$)
231[f]	207	7	207	68	239	14	238	13	33	τNN
	178	0	175	0	181	30	176	69	6	wC-C(ONHNH$_2$)
	175	0	169	20	168	2	169	2	7	δCCN (INH)
	156	20	166	9	158	139	166	62	11	IM
	138	28	139	2	153	131	150	222	15	τring (SAC)
	133	8	125	67	131	197	134	30	8	twSO$_2$, τring (SAC)
	128	28	119	140	123	173	130	44	10	IM
	109	2	106	28	119	58	108	306	13	τC-C (INH)
	109	52	99	33	106	118	105	6	9	IM
	89	8	95	15	106	2	96	41	17	IM
	87	1	83	8	101	76	92	2	19	IM
	82	1	82	8	94	2	84	2	12	IM
					84	93	79	74	5	IM
	67	3	64	116	77	181	73	64	12	IM
	61	0			68	28	67	105	7	IM
	47	4	32	1	54	380	49	131	22	IM
	27	8	30	1	33	225	43	117	16	IM
	13	5	10	1	29	1000	30	156	20	IM

[a] Wavenumbers (ῦ) in cm^{-1}; calculated infrared intensities (I_{IR}) in km mol^{-1}; calculated Raman scattering activities (a_R) in $Å^4$ u^{-1}; ν, bond stretching; δ, bending; γ, rocking; τ, torsion; w, wagging; tw, twisting; s, symmetric; as, anti-symmetric; IM, intermolecular mode; calculated wavenumbers were scaled as described in the Materials, and Experimental and Computational Methods section of this article. [b] Raman activities are normalized so that the activity of the highest band is 1000. [c] Predicted Davidov splitting (DS) for each mode was calculated as the difference between the highest and smallest calculated wavenumber among the four related modes of different symmetry. [d] Based on the vibration's animation mode of Chemcraft (G. A. Zhurko, Chemcraft - graphical program for visualization of quantum chemistry computations. Ivanovo, Russia, 2005. https://chemcraftprog.com). [e] Below 400 cm^{-1}: not-investigated. [f] Below 200 cm^{-1}: not-investigated.

Table S25. Assignment of the IR and Raman spectra for INH-SAC monohydrated salt (MH).[a]

MH Exp.		MH; Periodic B3LYP/6-31G(d,p)-D3					
		A_u		A_g			
ṽ (IR)	ṽ (Raman)	ṽ	I_{IR}	ṽ	a_R[b]	DS[c]	Assignment[d]
3570	3552	3578	1796	3572	85	6	νH_2O as
3498	3509	3460	2087	3468	172	7	νH_2O s
3337	3340	3400	114	3403	44	3	νNH_2 as
3275	3267	3279	355	3274	302	5	νNH_2 s
3223	3233	3222	3199	3224	162	3	νNH
3144		3106	98	3106	213	0	νCH (INH)
3084	3093	3101	22	3102	369	1	νCH (SAC)
		3101	60	3101	44	0	νCH (INH)
		3096	86	3096	169	1	νCH (SAC)
		3094	18	3095	165	1	νCH (SAC)
		3092	38	3092	52	0	νCH (INH)
	3083	3087	25	3086	91	1	νCH (SAC)
3067	3055	3073	274	3073	182	0	νCH (INH)
2507	2497	2576	10805	2552	289	24	νNH(···O=)
1667	1677	1676	191	1672	24	4	νC=O (INH)
1650		1654	307	1657	22	3	δNH(···O=)
		1643	211	1656	62	14	νCC (INH)
1638	1637	1635	875	1642	286	7	δNH_2
		1624	434	1618	34	6	νC=O (SAC), δH_2O
1607	1596	1617	1325	1612	33	5	δH_2O, νC=O (SAC)
1569	1575	1598	265	1596	68	2	νCC (SAC)
		1583	943	1586	97	3	νCC (SAC)
1549	1547	1550	348	1555	168	5	δNH
1525	1424	1528	54	1528	33	0	νCC (INH)
1494	1506	1500	312	1498	8	2	δCH (INH)
1456	1462	1453	108	1453	19	0	νCC (SAC)
1447		1441	46	1439	20	2	δCH (SAC)
1391	1387	1386	71	1386	24	0	νCC (INH)
1360	1367	1359	188	1359	31	0	νCC (SAC)
1345	1339	1339	77	1336	608	3	δCH (INH)
1322	1329	1319	17	1319	200	0	δCH (INH)
1295	1302	1300	535	1294	167	6	νCN (SAC)
		1292	33	1286	123	7	twNH_2
1259	1264	1282	187	1279	91	4	νCN (INH)
1248	1251	1248	146	1250	47	3	δCH (SAC)
1210	1215	1209	125	1209	52	1	δCH (INH)
		1194	230	1191	243	3	νCC (INH)
1182	1184	1184	839	1178	51	6	νSO_2 as

MH Exp.		MH; Periodic B3LYP/6-31G(d,p)-D3					
		A_u		A_g			
ṽ (IR)	ṽ (Raman)	ṽ	I_{IR}[b]	ṽ	a_R[b]	DS[c]	Assignment[d]
875	881	885	112	890	13	5	γCH (INH)
851	807	877	129	879	42	2	δring (INH)
		861	326	868	28	6	γCH (INH)
797	799	803	60	809	76	6	γNH
769	774	787	139	800	24	13	γCH (SAC)
757	743	757	240	755	33	1	δring (SAC)
724	720	740	209	748	27	8	τring (INH)
		735	42	735	28	0	τring (SAC)
702	711	696	8	732	27	36	δring (SAC)
		683	164	694	171	11	H_2O Rotation
678	673	676	21	674	35	2	δring (INH)
665	660	665	11	665	16	0	γC=O (SAC)
656	642	658	319	658	3	0	γC=O (INH)
646	611	650	8	650	77	0	δring (INH)
634		621	39	621	10	0	wC=O (SAC)
601	596	579	255	587	36	7	δSO_2
538	545	529	91	542	57	13	γSO_2
523	537	527	133	526	61	0	τring (SAC)
502	495	508	112	514	5	6	wSO_2
486		492	63	508	55	15	τring (INH)
478	461	466	306	495	26	29	H_2O Rotation
457		447	124	480	32	34	τring (INH)
446		442	45	450	17	8	τring (SAC)
434		432	444	438	19	6	H_2O Rotation
416		417	160	422	4	5	τring (INH)
407[e]	402	411	437	420	18	8	τring (INH)
		381	169	388	723	8	δring (SAC)
	383	373	192	381	153	8	δring (SAC)
	360	351	207	373	117	22	δOCN, δCNN (INH)
	336	333	226	350	30	17	δCNN, δOCN (INH)
	306	294	7	327	73	33	τring (SAC)
	283	267	12	289	23	22	τCN (SAC)
	261	258	70	287	41	29	twSO_2, τNN,
		254	53	263	45	9	γC-C(ONHNH_2)
	209[f]	239	63	249	222	10	τNN
		213	18	216	150	3	wC-C(ONHNH_2)
		191	24	201	102	10	δCCN (INH)
		184	58	191	217	7	IM

1142	1156	1155	68	1155	34	0	δCH (SAC)
		1138	57	1152	35	14	δCH (SAC)
		1131	276	1138	138	7	γNH(···O=)
1114	1114	1116	421	1120	113	4	νCC (SAC)
		1108	189	1115	55	8	νNN
1100	1106	1097	36	1099	32	2	νCC (INH)
	1075	1076	1285	1088	180	12	νSO_2 s
1051	1047	1069	174	1070	4	2	νCN (INH)
		1030	277	1027	14	3	νCS
1014	1017	1022	42	1020	198	2	νCC (SAC)
	1004	1009	7	1006	13	3	γCH (SAC)
988	990	998	82	999	149	1	δring (INH
		989	11	993	142	3	γCH (INH)
		980	10	979	88	1	γCH (SAC)
968		966	23	974	19	8	γCH (INH)
936	947	960	872	963	72	3	wNH_2
	902	902	34	908	9	6	γCH (SAC)
875	881	892	331	896	12	4	νNS

166	26	186	82	20	τring (SAC)
159	10	163	1000	4	twSO_2, τring (SAC)
152	36	159	315	7	τC-C (INH)
151	40	154	307	3	IM
146	33	142	79	3	IM
134	52	136	50	1	IM
121	127	127	51	5	IM
		124	111		IM
117	56	113	153	4	IM
99	33	102	630	3	IM
91	62	94	318	3	IM
80	35	89	396	9	IM
74	7	79	198	5	IM
65	4	69	75	4	IM
54	2	63	275	9	IM
		52	398		IM
		44	173		IM

[a] Wavenumbers ($\tilde{\upsilon}$) in cm^{-1}; calculated infrared intensities (I_{IR}) in km mol^{-1}; calculated Raman scattering activities (a_R) in $Å^4$ u^{-1}; ν, bond stretching; δ, bending; γ, rocking; τ, torsion; w, wagging; tw, twisting; s, symmetric; as, anti-symmetric; IM, intermolecular mode; calculated wavenumbers were scaled as described in the Materials, and Experimental and Computational Methods section of this article. [b] Raman activities are normalized so that the activity of the highest band is 1000. [c] Predicted Davidov splitting (DS) for each mode was calculated as the difference between the highest and smallest calculated wavenumber among the two related modes of different symmetry. [d] Based on the vibration's animation mode of Chemcraft (G. A. Zhurko, Chemcraft - graphical program for visualization of quantum chemistry computations. Ivanovo, Russia, 2005. https://chemcraftprog.com). [e] Below 400 cm^{-1}: not-investigated. [f] Below 200 cm^{-1}: not-investigated.

Table S26 - Cartesian coordinates (Å) for HCBS.

	x	*y*	*z*
8	2.419752000	2.106880000	0.124044000
8	-0.278922000	-0.560267000	1.020464000
8	-2.100186000	-1.873664000	-1.218749000
7	1.574648000	0.221438000	-0.832729000
1	1.685316000	-0.766021000	-1.014376000
7	0.278449000	0.680143000	-0.797158000
1	-0.019675000	1.295033000	-1.539718000
7	6.388842000	-0.995616000	0.227634000
6	2.576599000	0.953272000	-0.219728000
7	-2.629926000	-2.303613000	1.202370000
6	-0.587875000	0.260052000	0.170144000
6	-1.936473000	0.927170000	0.116265000
6	3.886892000	0.232065000	-0.076781000
6	3.988367000	-1.140487000	0.159796000
1	3.112938000	-1.769775000	0.272398000
6	-3.133973000	0.200314000	0.024514000
6	-2.001216000	2.316898000	0.242600000
1	-1.081691000	2.884458000	0.333562000
6	5.064205000	0.978599000	-0.138944000
1	5.020364000	2.048810000	-0.296577000
6	5.257346000	-1.699034000	0.308045000
1	5.365164000	-2.761618000	0.504823000
6	6.280674000	0.319575000	0.009238000
1	7.213323000	0.872992000	-0.046835000
6	-4.363783000	0.848189000	0.052776000
1	-5.268841000	0.262428000	-0.047760000
1	-3.342885000	-2.251927000	1.922011000
1	-1.724441000	-1.936997000	1.498289000
6	-4.412569000	2.236044000	0.187152000
1	-5.372288000	2.738775000	0.209518000
6	-3.234116000	2.968207000	0.283325000
1	-3.267585000	4.046059000	0.390293000
16	-3.130497000	-1.601432000	-0.223125000
8	-4.518183000	-2.005567000	-0.416617000

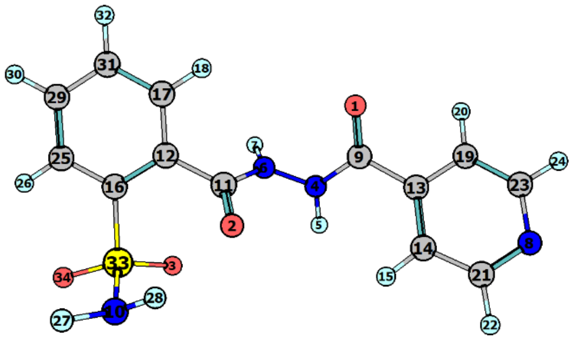

Table S27- Assignment of the IR and Raman spectra of HCBS.[a]

HCBS Exp.		HCBS B3LYP/6-311G++(d,p)				HCBS Exp.		HCBS B3LYP/6-311G++(d,p)			
$\tilde{\nu}$ (IR)	$\tilde{\nu}$ (Raman)	$\tilde{\nu}$	I_{IR}	I_R	Assignment[b]	$\tilde{\nu}$ (IR)	$\tilde{\nu}$ (Raman)	$\tilde{\nu}$	I_{IR}	I_R	Assignment[b]
3340		3618	58	3	νN6-H	969	965	984	0.3	0.5	γCH pyr
3320		3602	28	7	νN4-H	934	932	921	7	15	δCNN
3217	3281	3596	91	3	νNH_2 as	892	892	911	2	0.2	γCH ph
3165	3177	3444	107	6	νNH_2 s			906	9	0.3	δCNN
3096	3081	3204	6	13	νCH Ph			891	2	1	γCH pyr
3064	3073	3203	3	7	νCH Pyr	881	872	855	14	0.5	γCH pyr
		3194	6	13	νCH Ph	848		851	15	10	wNH_2
3016	3016	3182	3	6	νCH Ph	819	787	796	20	1	γCH ph
2963	2986	3181	6	8	νCH Pyr	798	764	773	51	0.2	τring ph
		3173	0.4	4	νCH Ph	787		768	4	0.5	τring pyr
2941	2945	3158	12	10	νCH Pyr	766		751	160	2	νSN
		3155	18	8	νCH Pyr	752	735	737	7	1	γC11=O
1680	1681	1763	149	16	νC9=O	742	719	725	41	1	γC9=O
1655	1659	1727	261	3	νC11=O	695	704	700	125	4	δring pyr
1621	1600	1632	8	14	νCC/CN pyr	670	665	690	111	1	δring ph
1590	1590	1629	4	14	νCCph			681	1	6	δring pyr
1575	1570	1608	3	5	νCC ph	657	654	662	12	10	δring ph
		1597	27	1	νCC/CN pyr	634	603	577	14	1	δC11=O
	1556	1591	70	1	δNH_2	592	591	567	38	2	δSO_2
1531	1528	1532	62	9	δNH-NH s	561	540	538	4	3	δCCN
1506	1501	1527	206	0.3	δNH-NH as	533	524	520	58	2.0	τring ph
		1512	116	1	δCH pyr, dN4H	496	491	480	71	4	γN6H
1485	1482	1495	50	0.1	δCH ph, dN6H	489		479	29	5.5	γN4H
1471	1457	1466	6	0.1	νCC ph	455	451	450	15	1.4	τS-N
1410	1409	1438	16	1	δCH pyr	443		434	17	5	δCCN
1341	1335	1354	2	1	δCH pyr	416	413	418	19	0.5	wSO_2
1332		1335	51	1	δSO2, νCC ph	404[c]	394	395	10	13	δC12-C11
1328		1325	117	8	νNN		382	387	2	2	δC13-C9, γN6H
	1324	1318	105	7	δSO_2, νCC ph			384	0.4	0	τring pyr
1295	1293	1291	1	0.1	δCH ph		360	367	11	1.1	τring ph
1278		1279	80	2	νCC/CN pyr		345	328	8	3	γph
1263	1278	1264	273	2	νCC/CN pyr		327	324	9	4	twSO_2
1223	1224	1245	4	3	δCH pyr			321	0.2	3	skeletal
1205	1206	1231	5	27	νNN, νC11C12		276	300	17	13	δCSN
1160	1171	1190	0.2	3	δCH ph		264	255	6	11	skeletal
	1160	1155	16	2	δCH ph			231	1	4	γSO_2
1137	1138	1136	60	3	γNH_2		204[d]	218	27	4	skeletal
		1127	115	2	νCS, δSO_2 s			192	10	5	skeletal
1114	1117	1116	41	4	δCH pyr			157	9	8	skeletal

1114	1117	1110	17	1	νCC/CN pyr	134	4	41	skeletal
1095	1098	1093	7	3	νCC/CN pyr	119	3	31	skeletal
1061	1062	1071	48	3	νNN, νC9N4	98	2	60	τCS
1048	1039	1059	6	5	νCC ph	87	3	32	skeletal
		1051	12	15	δring ph, νCS	57	1	114	τC12C11
1022	1014	1016	0.1	0.1	γCH ph	47	3	187.9	τC9C13
1004	1000	1011	2	21	δring pyr	26	1	137	skeletal
996		1006	1	0.04	γCH pyr	21	2	468.8	τNN
969	965	989	2	0.1	γCH ph	15	4	1000.0	skeletal

[a] Wavenumbers (ῡ) in cm^{-1}; calculated infrared intensities (I_{IR}) in km mol^{-1}; calculated Raman scattering intensities (I_R) were normalized to 1000; ν, bond stretching; δ, bending; γ, rocking; τ, torsion; w, wagging; tw, twisting; s, symmetric; as, anti-symmetric; ph, phenyl ring: pyr, pyridyl ring. [b] Based on the vibration's animation mode of Chemcraft (G. A. Zhurko, Chemcraft - graphical program for visualization of quantum chemistry computations. Ivanovo, Russia, 2005. https://chemcraftprog.com). [c] Below 400 cm^{-1}: not-investigated. [d] Below 200 cm^{-1}: not-investigated.

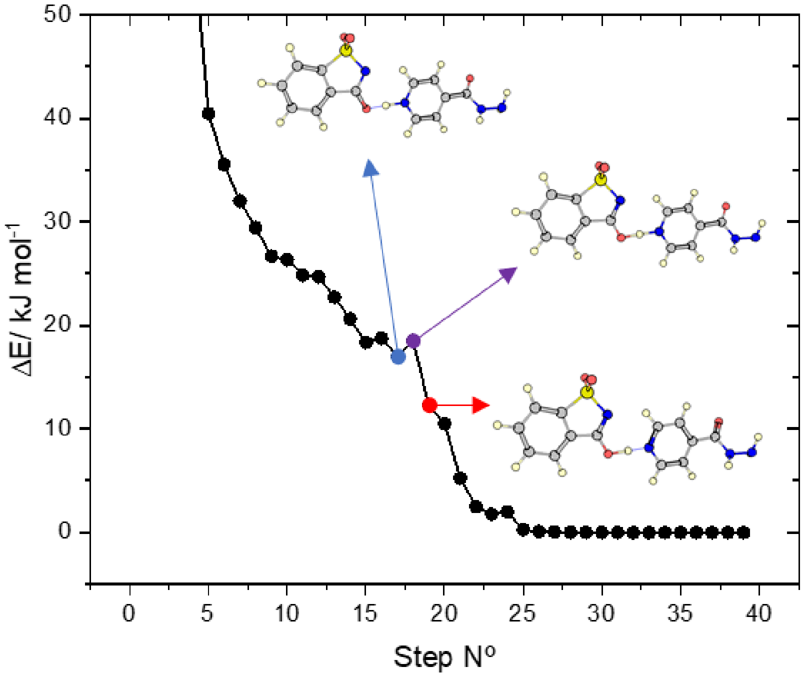

Figure S1 – Change of energy along geometry optimization for the isoniazid/saccharin isolated dimer. Starting structure corresponds to $(INH{+}H)^{+}/(SAC{-}H)^{-}$, and final structure to INH/SAC-OH. Step Nº 17 corresponds to the last saved optimization geometry before this proton relocation is completed. Zero of energy corresponds to the calculated minimum energy structure.

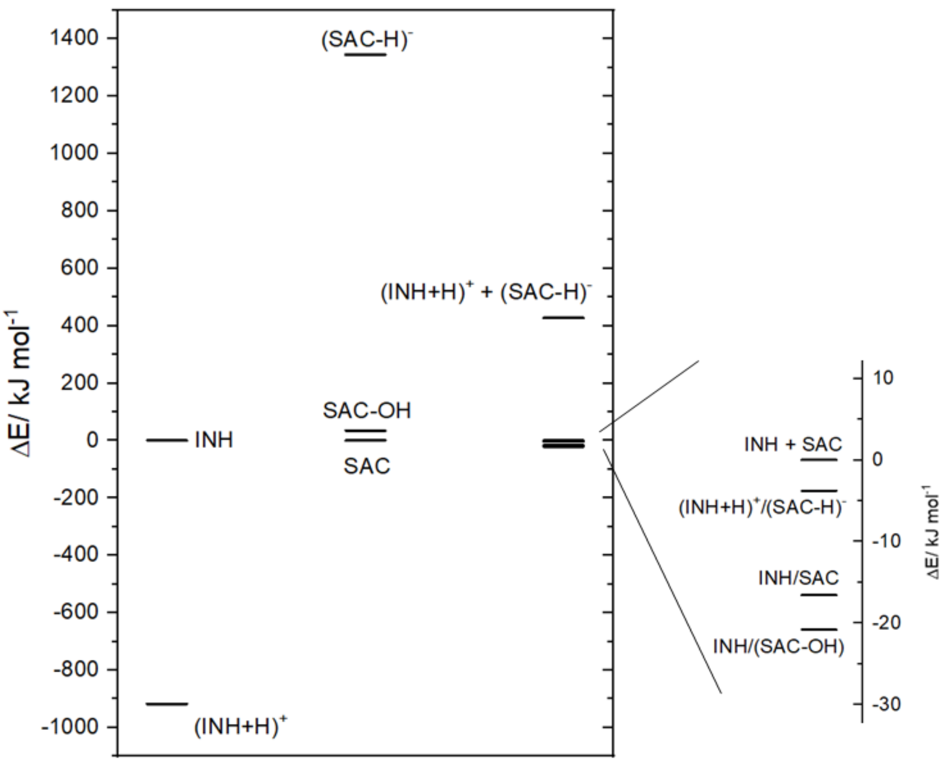

Figure S2 – Calculated B3LYP/6-311++G(d,p) relative energies (zero-point corrected) of the different INH and SAC isolated systems investigated in this study. The $(INH{+}H)^{+}/(SAC{-}H)^{-}$ structure is not a minimum and converts to INH/SAC-OH upon optimization (see Figure S1).

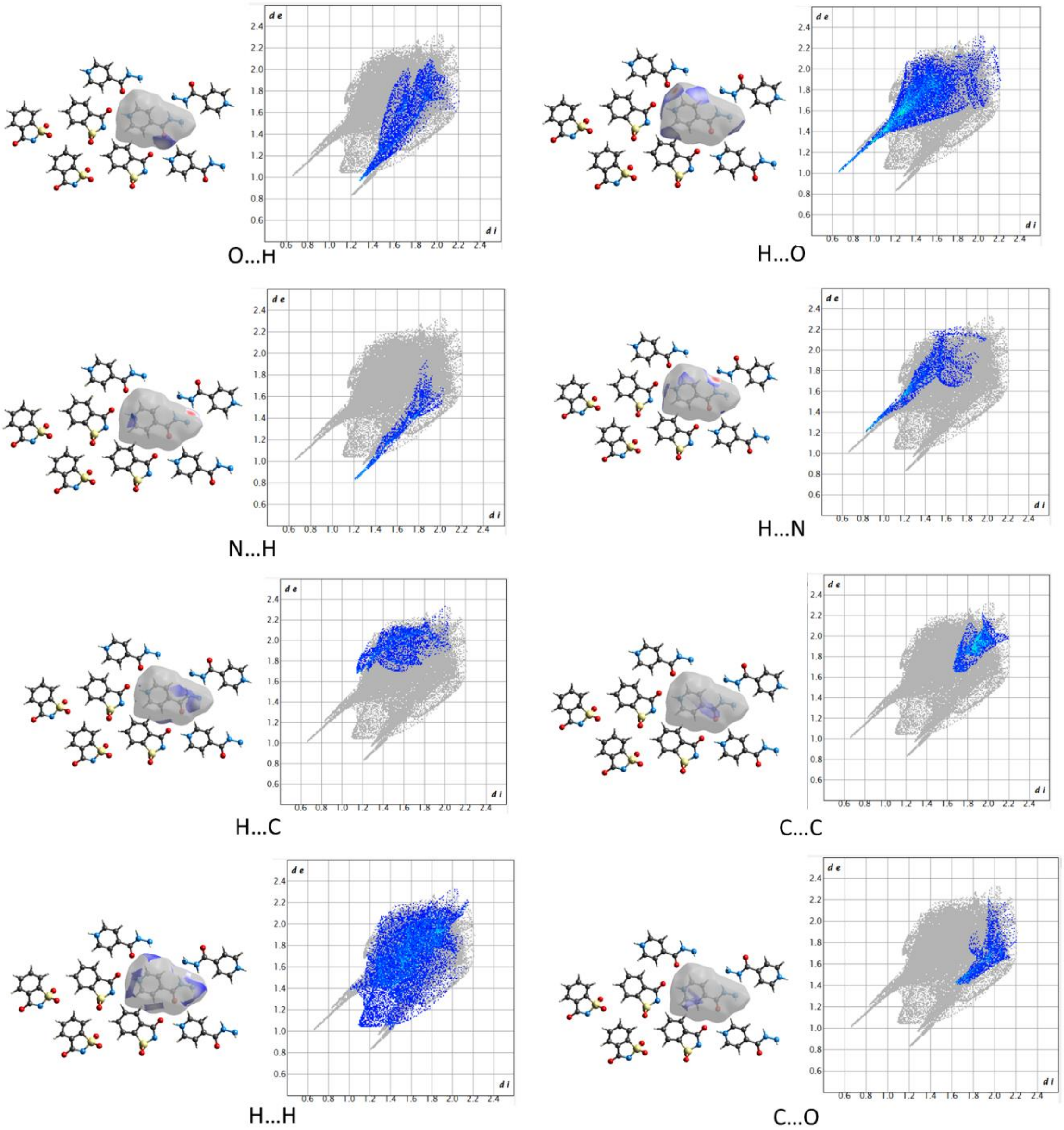

Figure S3 – Hirshfeld surface for the $(INH{+}H)^{+}$ ion in the $(INH{+}H)^{+}/(SAC{-}H)^{-}$ salt (A) and 2D fingerprint plots for the different contacts.

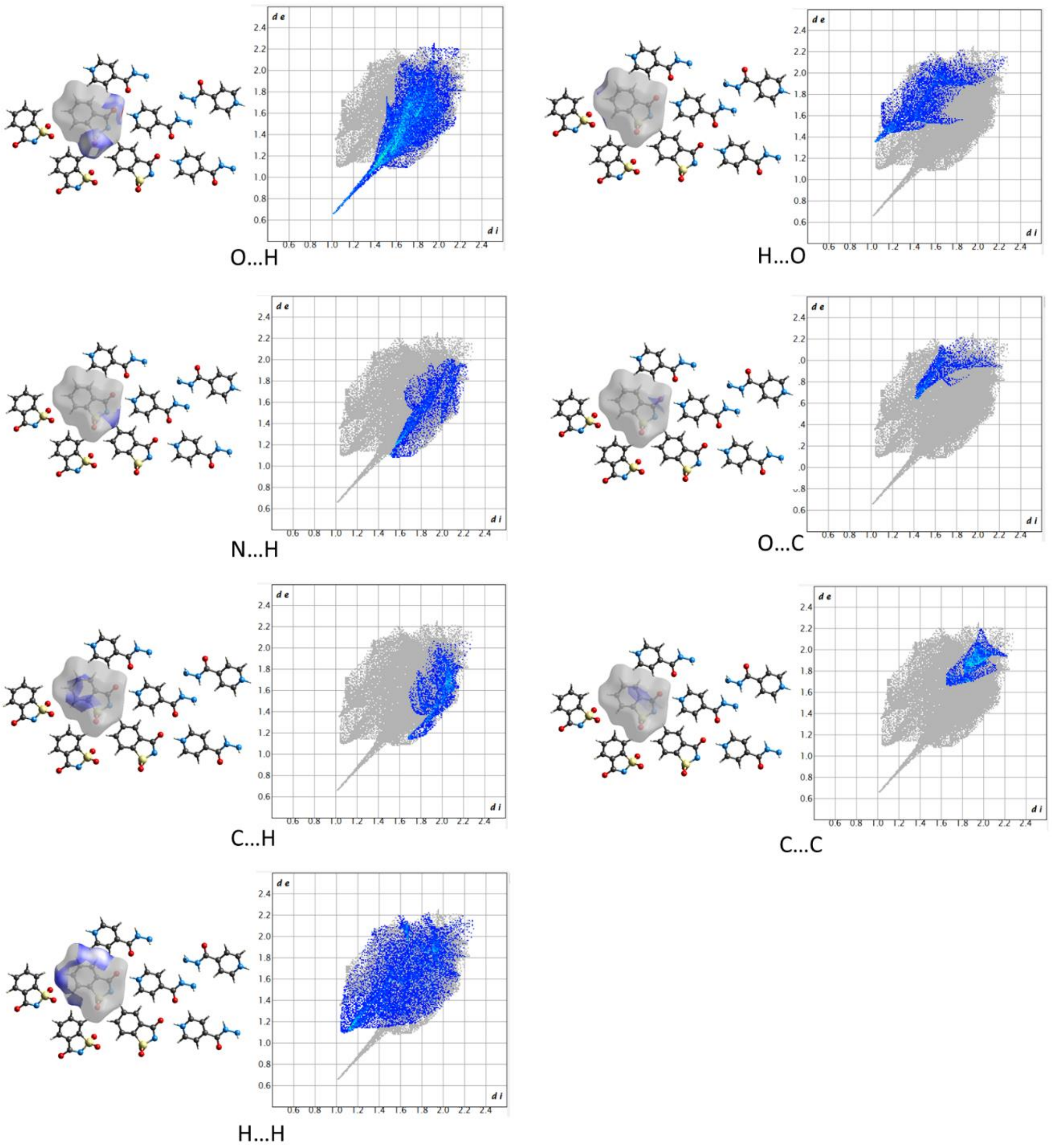

Figure S4 – Hirshfeld surface for the (SAC–H)$^{-}$ ion in the (INH+H)$^{+}$/(SAC–H)$^{-}$ salt (A) and 2D fingerprint plots for the different contacts.

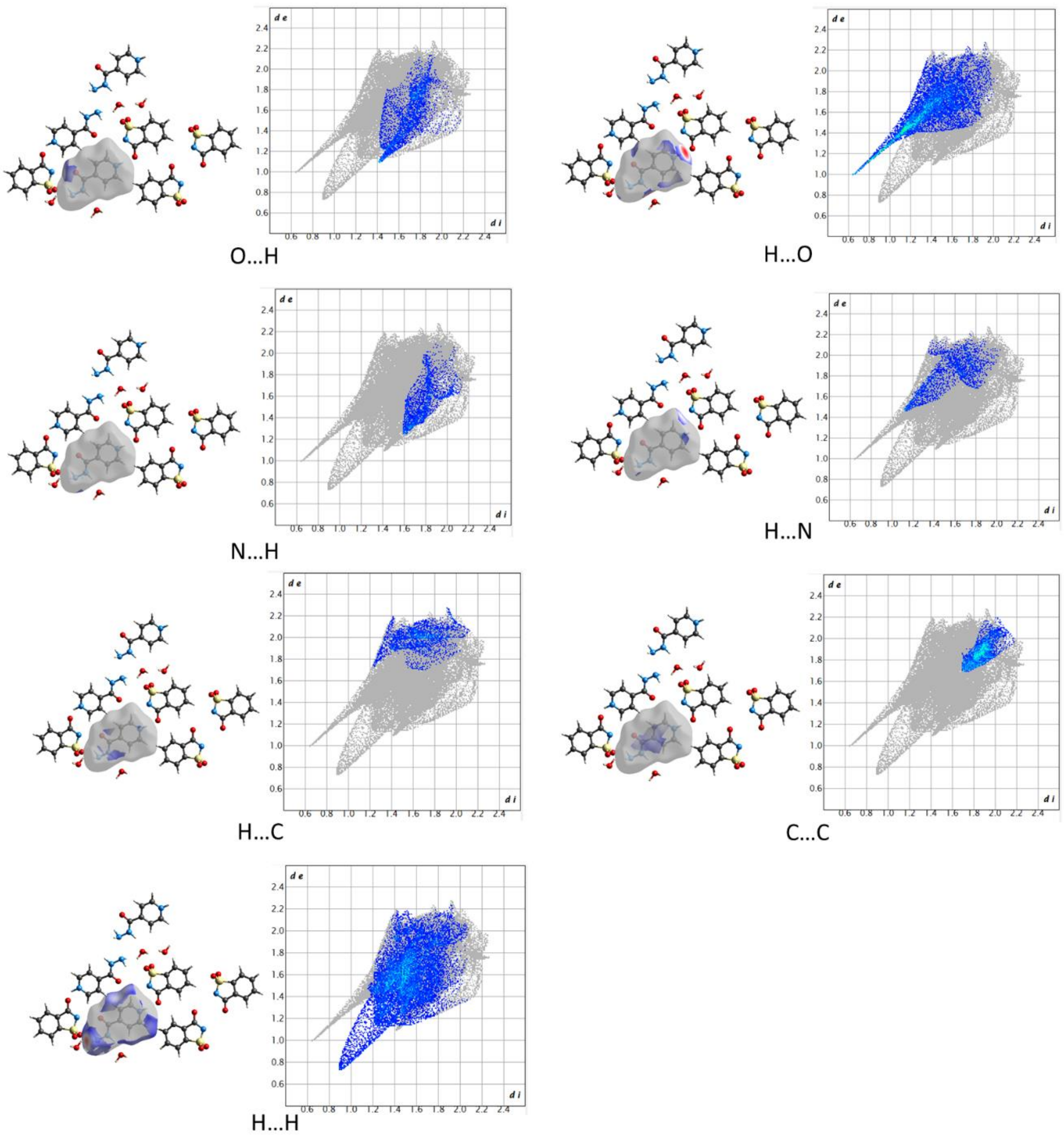

Figure S5 – Hirshfeld surface for the $(INH+H)^+$ ion in the $(INH+H)^+/(SAC–H)^-.H_2O$ salt (MH) and 2D fingerprint plots for the different contacts.

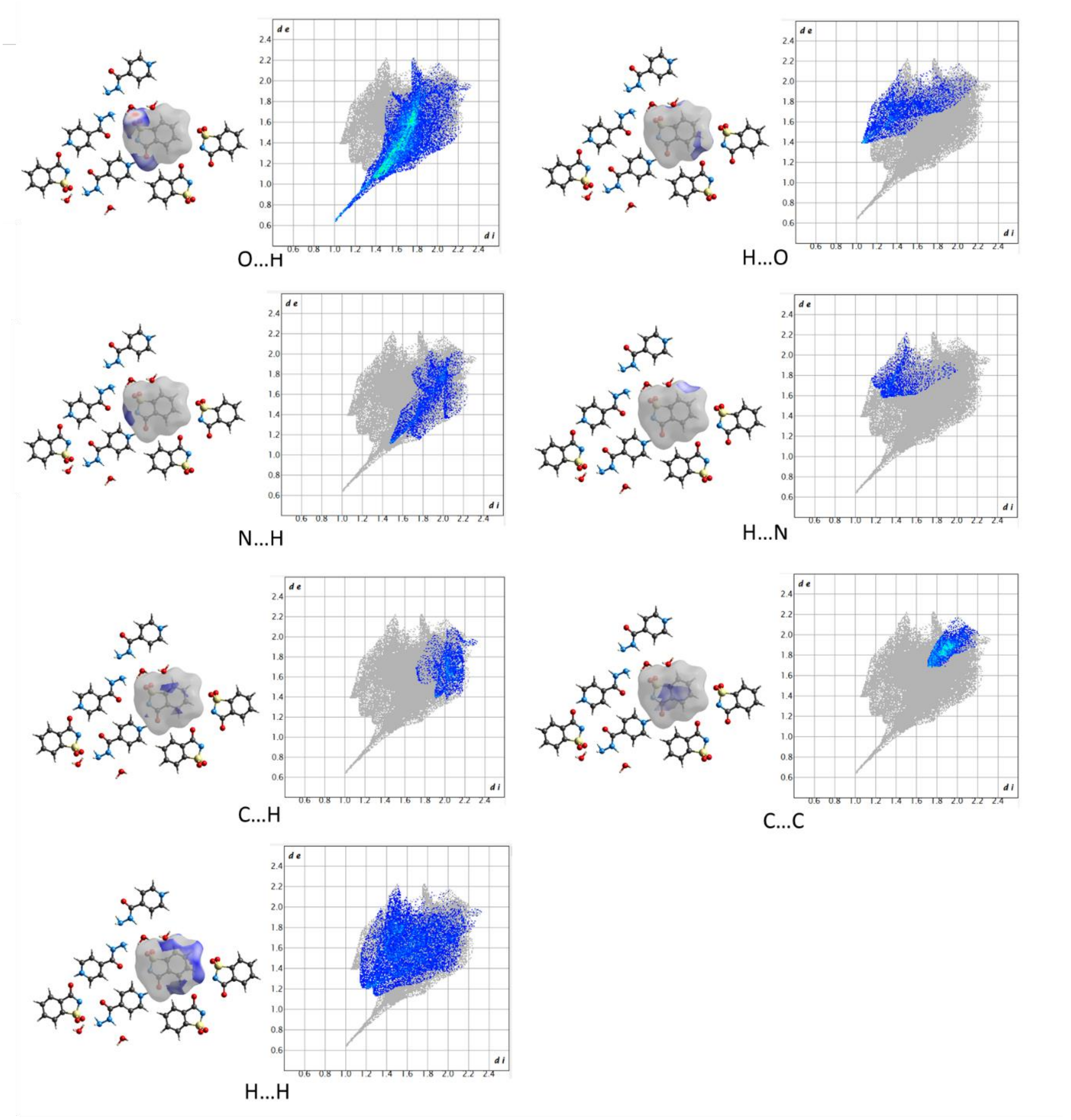

Figure S6 – Hirshfeld surface for the (SAC–H)$^{-}$ ion in the (INH+H)$^{+}$/(SAC–H)$^{-}$.H$_2$O salt and 2D fingerprint plots for the different contacts.

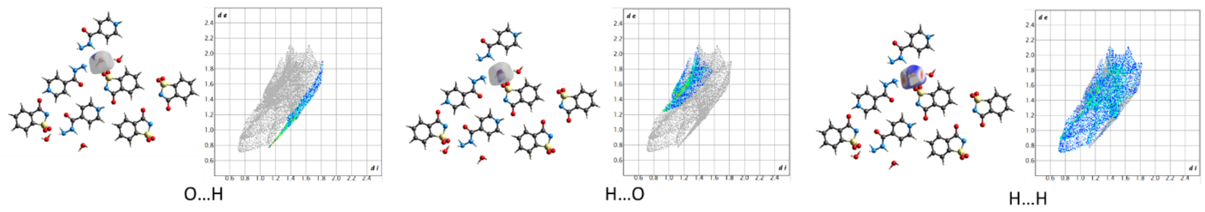

Figure S7 – Hirshfeld surface for the water molecule in the (INH+H)$^{+}$/(SAC–H)$^{-}$.H$_2$O salt and 2D fingerprint plots for the different contacts.

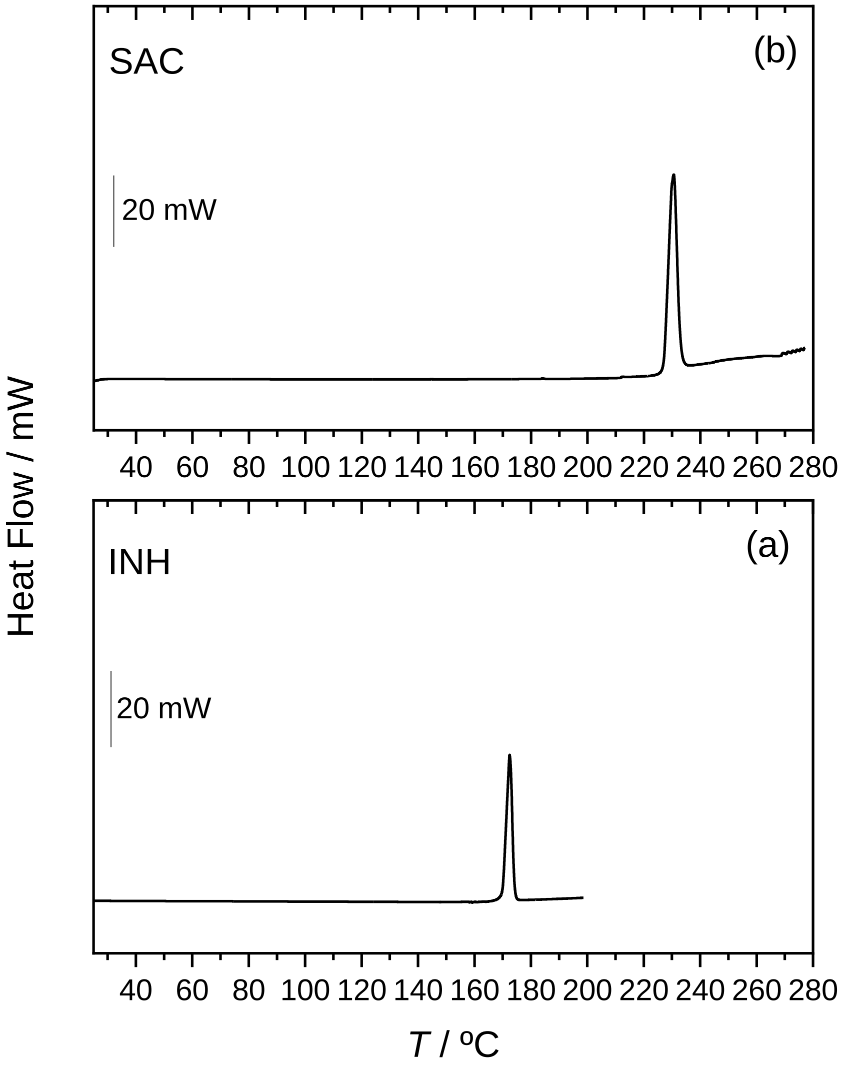

Figure S8 - DSC curves of (a) pure INH (polymorph 1), and (b) pure SAC. m_{INH} = 5.03 mg; m_{SAC} = 7.64 mg. β = 10 ºC min^{-1}.

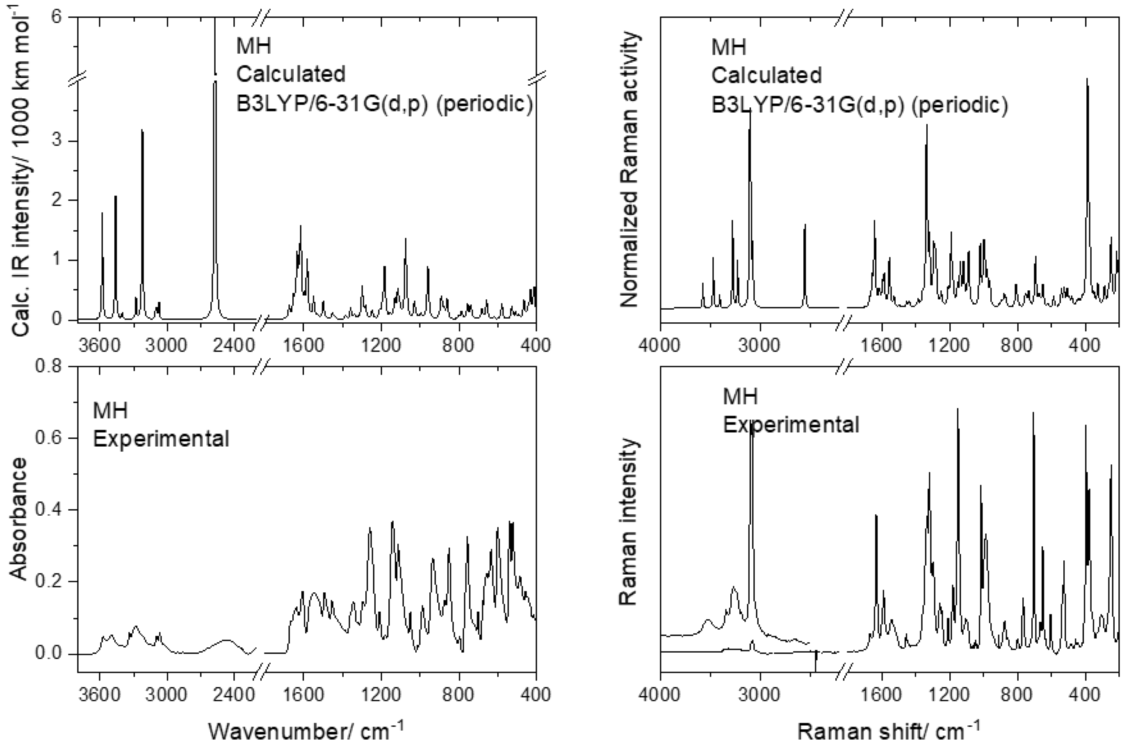

Figure S9 – Experimental and calculated (B3LYP/6-31G(d,p)-D3; periodic) infrared (*left*) and Raman (*right*) spectra of MH.

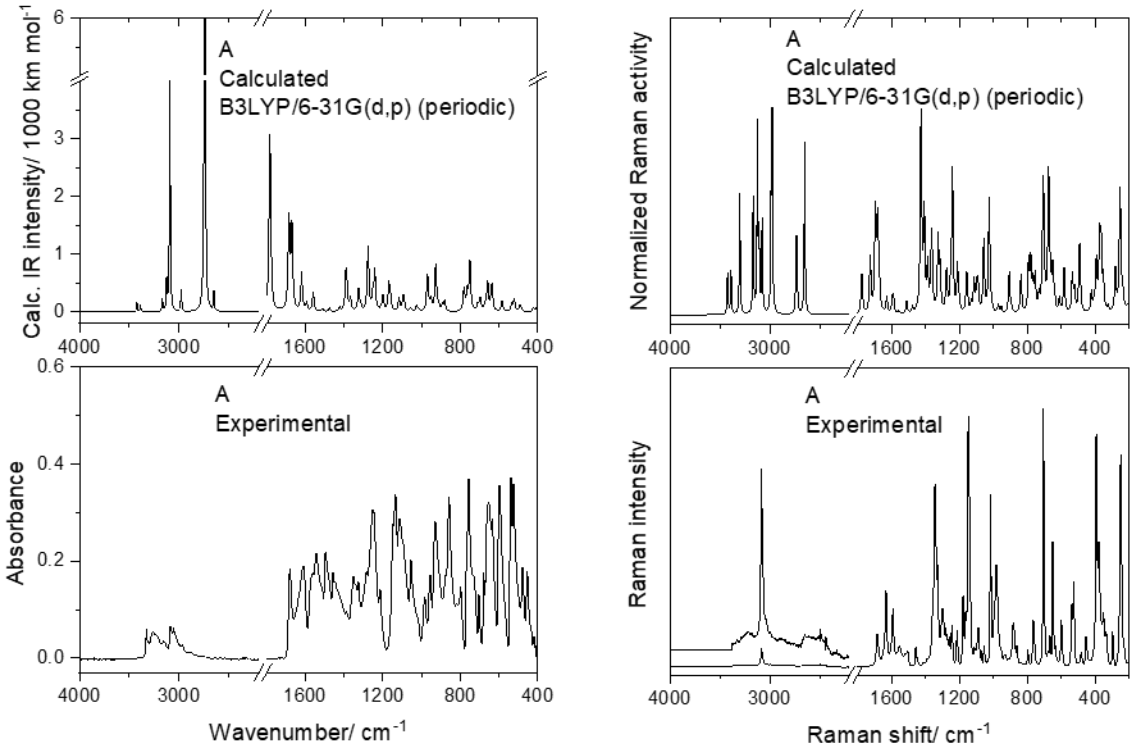

Figure S10 – Experimental and calculated (B3LYP/6-31G(d,p)-D3; periodic) infrared (*left*) and Raman (*right*) spectra of A.
